# 3D printing of bio-instructive materials: Toward directing the cell

**DOI:** 10.1016/j.bioactmat.2022.04.008

**Published:** 2022-04-23

**Authors:** Piotr Stanisław Zieliński, Pavan Kumar Reddy Gudeti, Timo Rikmanspoel, Małgorzata Katarzyna Włodarczyk-Biegun

**Affiliations:** a Polymer Science, Zernike Institute for Advanced Materials, University of Groningen, Nijenborgh 4, 9747 AG, Groningen, the Netherlands; b Biotechnology Centre, The Silesian University of Technology, B. Krzywoustego 8, 44-100, Gliwice, Poland

**Keywords:** Tissue engineering, Additive manufacturing, Melt electrowriting, 3D scaffold, Cell differentiation, Biomaterials

## Abstract

Fabrication of functional scaffolds for tissue engineering and regenerative medicine applications requires material systems with precise control over cellular performance. 3D printing is a powerful technique to create highly complex and multicomponent structures with well-defined architecture and composition. In this review paper, we explore extrusion-based 3D printing methods (EBP, i.e., Near Field Electrospinning (NFES), Melt Electrowriting (MEW), Fused Deposition Modeling (FDM), and extrusion bioprinting) in terms of their ability to produce scaffolds with bio-instructive properties. These material systems provide spatio-temporal guidance for cells, allowing controlled tissue regeneration and maturation. Multiple physical and biochemical cues introduced to the EBP scaffolds are evaluated in their ability to direct cell alignment, proliferation, differentiation, specific ECM production, and tissue maturation. We indicate that the cues have different impacts depending on the material system, cell type used, or coexistence of multiple cues. Therefore, they must be carefully chosen based on the targeted application. We propose future directions in bio-instructive materials development, including such concepts as metamaterials, hybrid living materials, and 4D printing. The review gathers the knowledge essential for designing new materials with a controlled cellular response, fabrication of advanced engineered tissue, and developing a better understanding of cell biology, especially in response to the biomaterial.

## Highlights

• Extrusion-based printing allows for the fabrication of bio-instructive scaffolds.
• Physical cues mostly direct cell alignment and spatial distribution.
• Biochemical cues are used to drive cell differentiation in a system-specific way.
• We extensively describe new possibilities in bio-instructive materials printing.

## Graphical abstract

**Figure:**
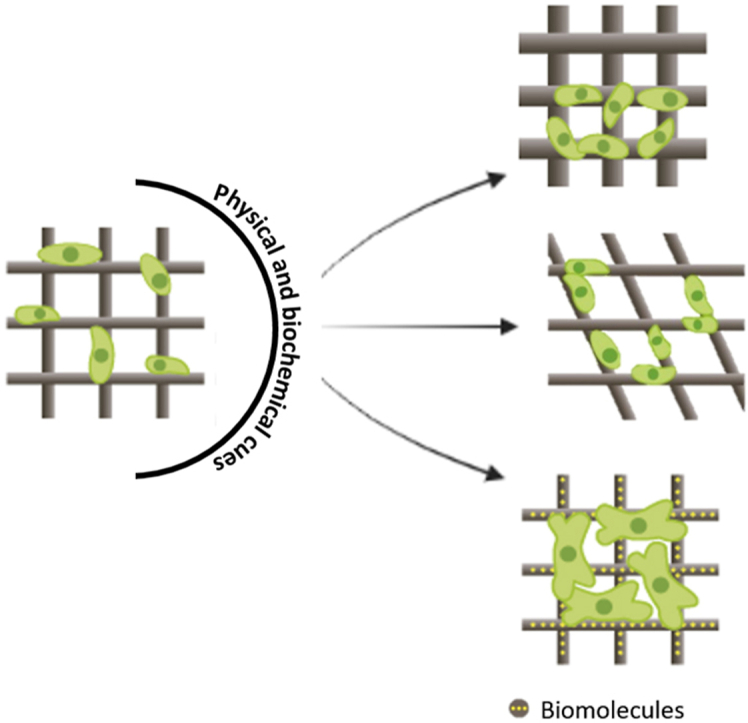

## Abbreviations

adECM: adipose-decellularized extracellular matrix
ADMSCs: adipose tissue-derived mesenchymal stem cells
aHSCs: primary fetal activated hepatic stellate cells
algMC: alginate methylcellulose
ALP: alkaline phosphatase
BdECM: bone decellularized extracellular matrix
BDNF: brain-derived neurotrophic factor
BG: bioglass
BMP-2: bone morphogenetic protein-2
BMSCs: bone marrow stem cells
CaP: calcium phosphate
CaSH: calcium sulfate hemihydrate
CChon: costal chondrocytes
cdECM: cartilage-decellularized extracellular matrix
ciPTECs: human conditionally immortalized proximal tubular epithelial cells
CT: Citroflex
CMs: neonatal rat cardiomyocytes
CNF: nanocellulose
COL1: collagen type I
ColMA: methacrylate collagen
CPCs: cardiac progenitor cells
CS: composite scaffolds
DAP: decellularized adipose tissue
DCM: dichloromethane
dECM: decellularized extracellular matrix
Dex: dexamethasone
EBP: extrusion-based 3D printing
ECM: extracellular matrix
EPL-: ε-poly-l-lysine
FDM: Fused Deposition Modeling
FEM: finite element method
FGelMA: fish gelatin meth-acrylamide
GAG: glycosaminoglycan
g-C₃N4: graphitic carbon nitride nanoparticles
GEL-: gelatin
GelMA: gelatin methacrylate
GO: graphene oxide
HA: hyaluronic acid
HaCaTs: human keratinocytes
hAD-MSCs: human adipose tissue-derived mesenchymal stem cells
HAp-: hydroxyapatite
hBMSCs: human bone marrow stem cells
hdECM: heart-decellularized extracellular matrix
HDFs: dermal fibroblasts
HDMECs: human dermal microvascular endothelial cells
HFIP: 1,1,1,3,3,3-hexafluoro-2-propanol
hMF: human mammary fibroblasts
hTMSCs: human inferior turbinate-tissue-derived mesenchymal stromal cells
HUVECs: human umbilical vein endothelial cells
hUVSMCs: human umbilical cord vein smooth muscle cells
IGF-1: insulin-like growth factor-1
iPSC-CM: human-induced pluripotent stem cell-derived cardiomyocytes
KGN: kartogenin
L6: rat myoblast cells
L929: mouse fibroblast cells
Lx2: human hepatic stellate cell line
MC3T3-E1: murine calvarial pre-osteoblast cells
MECM: meniscus extracellular matrix
MEW: Melt Electrowriting
mMSCs: mouse mesenchymal stem cells
MPs: milk proteins
MSCs: mesenchymal stem cells
MA: mucic acid
NFES: Near Field Electrospinning
nHAp-: nano-hydroxyapatite
NHDFs: neonatal human dermal fibroblasts
NHLFb: primary human lung fibroblasts
nnHAp-: nano-needle hydroxyapatite
OCT2: transporters organic cation transporter 2
PANI: polyaniline
PCL-: polycaprolcatone
PCSA: peptide-conjugated sodium alginate
PcycloPrOx: poly(2-cyclopropyl-2-oxazoline)
PDA: polydopamine
PCEC: poly (ε-caprolactone)-poly (ethylene glycol)
PEEK: polyetheretherketone
PU: poly(ester)urethane
PGA: poly(glutamic acid)
pHAp-: plate hydroxyapatite
PHBH: poly(3-hydroxybutyrate-*co*-3-hydroxyhexanoate)
PHB: poly(3-hydroxybutyrate)
pHCM: primary human cardiomyocytes
pHMGCL-: poly(hydroxymethylglycolide-*co*-ε-caprolactone)
PLA: poly(lactic acid)
PLCL-: poly (l-lactic acid-ε-caprolactone)
PLGA: poly(D, l-lactide-*co*-glycolide)
PLL-: poly-l-lysine
PLLA: poly(l-lactic acid)
PMMA: poly(methyl methacrylate)
PnPrOx: poly(2-*n*-propyl-2-oxazoline)
Pred: prednisolone
PRP: human platelet-rich plasma
PVA: poly(vinyl alcohol)
PVAc: poly(vinyl acetate)
rBMSCs: rat bone marrow stromal cells
RCSA: alginate precursor with conjugates of RGD
rGO: reduced graphene oxide
rMSCs: rabbit mesenchymal stem cells
ROS: reactive oxygen species
SES: solution electrospinning
SF: silk fibroin
SMP: silk microparticles
SMSCs: synovium-derived mesenchymal stem cells
SN: Syncroflex
SNF: silk nanofibers
SrHAp-: strontium substituted hydroxyapatite
TdECM: tendon-derived decellularized extracellular matrix
T_g_: glass transition temperature
T_m_: melting temperature
VEGF: vascular endothelial growth factors
WF: whey protein
YCSA: alginate precursor with conjugates of YIGDR
YIGDR: tyrosine-isoleucine-glycine-serine-arginine
β-TCP: β-tricalcium phosphate

## 1 Introduction

The main non-cellular component of a native tissue is an extracellular matrix (ECM). ECM is a 3-dimensional water-based network of proteins and proteoglycans, produced and remodeled constantly by the cells [1]. ECM contains structural, biochemical, and biomechanical signaling domains – the cues necessary for cell adhesion, migration, proliferation, and differentiation [2]. Every tissue has a specific hierarchical organization, with a certain combination of the signaling molecules [3], mechanical properties, and embedded cells [4].

As ECM provides a natural environment and support for the cells, tissue engineering approaches aiming to reconstruct native tissues commonly employ material scaffolds. The scaffold should constitute a suitable niche for cells to proliferate, differentiate, and ultimately form the new functional tissue [5,6]. Therefore, the ideal scaffold needs to meet a few key requirements. (I) Scaffold must be non-toxic, biocompatible, and bioactive [7], which means that the supporting cells should maintain their original morphology and function, migrate, and proliferate without eliciting inflammatory responses [8]. Upon implantation, good integration with surrounding tissues should be ensured [9]. (II) It should mimic the native structural arrangement of ECM fibers in a cell- and tissue-specific manner [10], which can be achieved with an appropriate scaffold architecture and surface topography [7]. These factors are responsible for the initial distribution of cells and influence cellular response and further tissue formation. (III) Another critical factor related to the architecture is open porosity. Interconnected pores allow selective permeability and transport of the oxygen, nutrients, waste products, and cell migration within the construct. Optimal pore size allows vascularization of newly formed tissue [11]. (IV) The scaffold should also have sufficient mechanical stability to support the growing cells and mimic mechanical properties of the specific native tissue, such as stiffness or strength [12]; e.g., it is known that softer substrates favor neuron spreading [13]. Additionally, the stability allows good surgical handling during implantation [14]. (V) Moreover, the degradation time needs to be adjusted to the specific application; for example, the degradation between 4 and 6 weeks is required for engineered skin tissue [[14], [15], [16]].

**Fig. 1:**
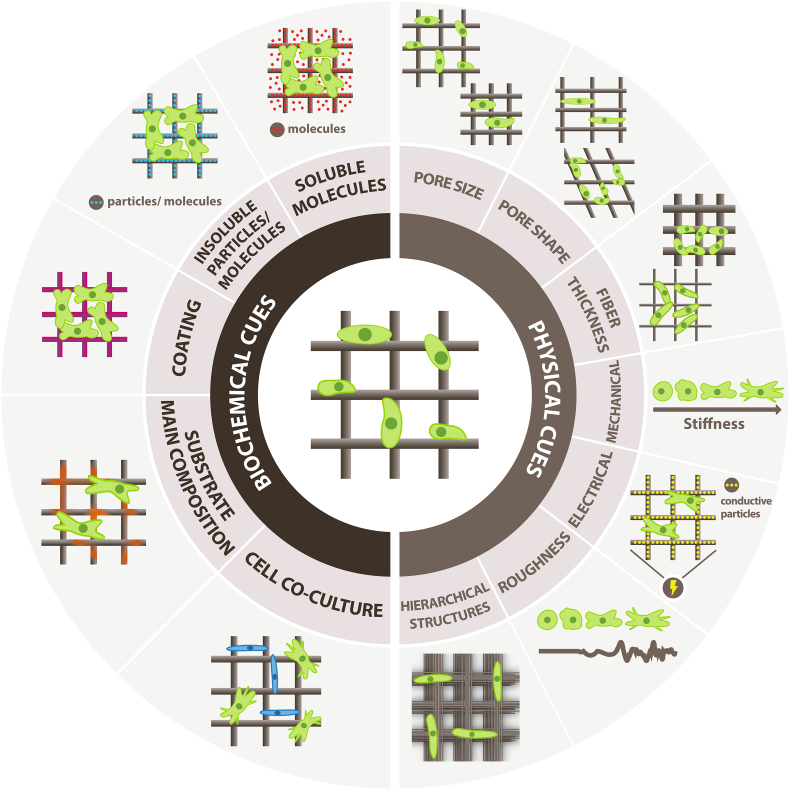
Schematic representation of biochemical and physical cues for cells provided by the printed scaffold.

Meeting all of those requirements in one material system does not guarantee the successful formation of the complex and hierarchical structure recapitulating living tissues. Therefore, the concept of bio-instructive materials that can precisely control cell performance by providing specific physical and biochemical cues and direct tissue formation and its proper function gains much interest [9]. Bio-instructive materials provide spatio-temporal guidance for cells in a 3D environment [17] by introducing cell signaling [18] to closely imitate specific mechanical, biological, and compositional properties of native tissues. The cues can be presented inside the scaffold matrix or on the scaffold surface, and they can be divided into physical and biochemical ones (Fig. 1). Physical cues include mechanical, electrical, or topographical stimuli such as roughness or hierarchically ordered structures; biochemical signals include specific drugs, proteins, growth factors, or incorporated insoluble particles [19]. They direct cell behavior by regulating the adhesion, proliferation [20], migration patterns [21], differentiation of stem cells [22]. Both types of cues can be combined in one material system for better performance.

This review describes the strategies for obtaining cell guiding and bio-instructive materials based on the EBP techniques. 3D printing provides unprecedented control over spatial distribution and patterning of different materials, allowing for the independent incorporation of different cues. For example, material stiffness, porosity, and composition can be tuned precisely and independently to match targeted tissues; also, gradients in the signaling can be relatively easily incorporated. Employing EBP for bio-instructive materials is a powerful growing approach, allowing to reach complexity relevant for building close mimics of native tissues. Here, we discuss different physical and biochemical cues, methods of their incorporation via printing, and applications of bio-instructive printed materials. Finally, future research perspectives are presented.

## 2 Extrusion printing for bio-instructive materials

### 2.1 Printing techniques

The principle of 3D printing is to precisely deposit the material (ink) to obtain a well-defined 3D structure. Components such as cell-laden solutions, biomaterials, and biological molecules can be deposited with high precision in three dimensions (x, y, and z) [5,23]. This enables building complex, hierarchical, and specific 3D scaffolds containing multiple cues that mimic defined features of the native microstructures of the tissues and organs [24].

**Fig. 2:**
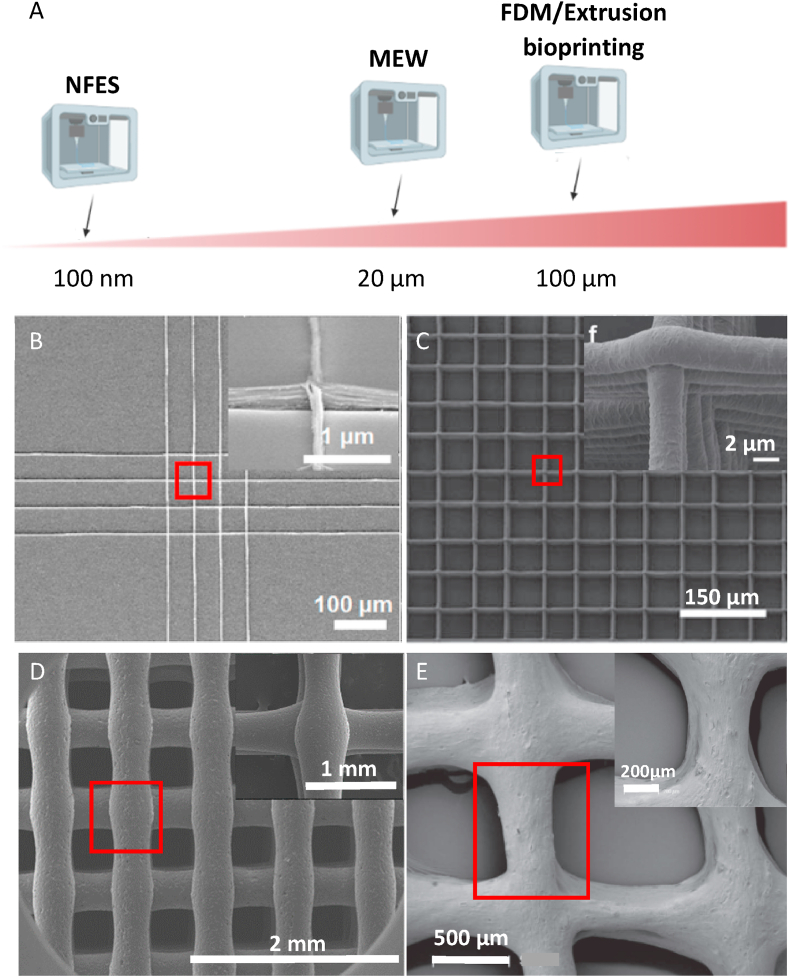
Resolution of EBP techniques. (A) Schematics showing typical strand sizes obtained with different EBP approaches. Typical scaffold printed with (B) NFES, (C) MEW, (D) FDM, and (E) extrusion bioprinting. (B) Reproduced with permission from Ref. [52], Copyright © 2019 American Chemical Society. (C) Adapted under the terms of the Creative Commons Attribution 3.0 license from Ref. [55], Copyright © 2020 IOP Publishing Ltd. (D) Adapted under the terms of the Creative Commons Attribution License from Ref. [56], Copyright © 2018 MDPI. (E) Adapted with permission from Ref. [57], Copyright © 2017 IOP Publishing Ltd.

Various 3D printing techniques are used to fabricate scaffolds [25], with EBP approaches being the most broadly explored. EBP gained interest due to the relatively good resolution, a wide range of material choices, the possibility of high cell density printing, and affordability [26]. Moreover, it enables efficient printing of large, medically relevant scaffolds [27,28]. The most commonly used methods that can be classified as EBP are extrusion bioprinting, Fused Deposition Modeling (FDM), Melt Electrowriting (MEW), and Near Field Electrospinning (NFES) (Fig. 2) [29].

This review associates extrusion bioprinting with hydrogel-based materials [30]. The material is placed in a reservoir and deposited on the printing stage with pressure or a mechanically-driven system [31]. For hydrogel printing, usually a crosslinking agent is required; a homogeneity of crosslinker distribution needs to be ensured [30]. The advantage of extrusion bioprinting is the possibility of adding the cells directly to the ink. Such a mixture composed of biomaterial and cells is called bioink [32]. The size of nozzles for printing bioinks is typically in the range of 150 μm – 600 μm to reduce high shear stresses and avoid clogging of the needle and cell death [33]. Hydrogels with different viscosities can be printed (between 30 and 6⋅10⁷ mPa⋅s [34]), and scaffolds in centimeter-scale are easily obtained [35].

While extrusion bioprinting is mainly used for printing hydrogel scaffolds with embedded cells, in the FDM process, the melted thermoplastic material is extruded onto the printing platform [36,37]. The material can be provided in the form of ready filament or pellets (depending of the printer), and is heated above melting temperature (T_m_) to enable the flow through the nozzle [38]. To allow quick solidification, materials with a relatively high glass transition temperature (T_g_) are required [39,40]. During the printing process, the temperature of the already deposited layer, in contact with the freshly printed layer, increases above the T_g_, allowing good bonding between them. The low T_g_ carries a risk of disturbances in structure and non-uniform strength of the interlayer connections [41]. The FDM printers usually utilize nozzles with 0.1 mm – 1 mm diameter resulting in a maximum strand resolution of around 100 μm. Printing scaffolds in centimeter- [39] or even meter-scale for industrial applications is possible [42].

MEW offers higher resolution printing. This novel biofabrication method combines electrospinning and 3D printing principles, taking advantage of both techniques. With the assistance of the air pressure, the electrical field is used to draw fibers of a molten polymer through a metal nozzle onto a computer-controlled collector plate. By repetitive fiber-by-fiber stacking, highly complex and precise 3D constructs are obtained [43,44]. Typically, fibers printed with the MEW technique are in the range of a couple of micrometers (2 μm – 50 μm) [29,45]. After careful optimization, a fiber diameter below 1 μm can be obtained [46]. The scaffold's quality is dependent on the multiple printing parameters: applied voltage and pressure, collector speed, working distance, and process temperature. MEW allows printing volumetric scaffolds up to ca. 7 mm in height, with more than 300 accurately deposited layers [47].

NFES is the EBP approach with the highest resolution. It requires a higher voltage and shorter distance between nozzle and collector plate than MEW [48] and utilizes polymer melt or solution [49]. In traditional electrospinning, the fibers are randomly deposited on the collector due to the bending instabilities of the jet. In NFES, instabilities are overcome by reducing the spinning distance and applied voltage. It allows the control over electrospun fiber deposition on the moving platform, which results in the fabrication of 3D well-aligned nanofiber scaffolds [49]. NFES gives a sub-100nm resolution and fibers’ thickness below 20 nm using solution-based inks [50,51]; the lowest reported fiber diameter of the melted material was ca. 700 nm [49]. The NFES is a low-cost process with high controllability up to 80 printed layers (height of up to a couple of micrometers) [52]; different patterns can be obtained, such as triangles, diamonds, or hexagons [53]. The downside of producing ultrathin nanofibers is using organic solvents that are often toxic [54].

### 2.2 Materials

Depending on the processing principles of the specific EBP approach, different biomaterials are used. The hydrogels are associated in this review with extrusion bioprinting. The main advantages of hydrogel-based biomaterials include their biocompatibility, resemblance of hydrated tissue environment, biodegradation, and the possibility of cell encapsulation prior to printing [[58], [59], [60]]. Many natural and synthetic hydrogels, and their combinations, have been used to produce bio-instructive scaffolds [[61], [62], [63], [64]]. Most prominent examples of natural hydrogels are collagen [65], gelatin [66], alginate [67], chitosan [68] or decellularized extracellular matrix (dECM) [[69], [70], [71]]. Synthetic materials for extrusion printing include gelatin methacrylate (GelMA), polyacrylamide, or poly(ethylene glycol) [[71], [72], [73]]. Naturally derived inks are typically nontoxic, and often benefit from the presence of ECM proteins and adhesion domains for cells; however, they are weak by nature, and their properties can be tailored only to a certain extent [70,71]. The mechanical properties of synthetic hydrogels can usually be more flexibly adapted. Yet, those materials lack adhesion sites, thus showing lower cell adhesion and proliferation than natural ones [71]. To improve the properties of hydrogels in terms of biological performance and mechanical stability after printing, chemical modifications can be introduced, or multiple hydrogel types can be mixed [65,67,74,75].

FDM approach utilizes thermoplastic materials, which are often heated up to high temperatures (over physiological range), and melted before or during the deposition process. As a result, cell encapsulation is not possible; however, these materials typically offer better mechanical performance [76]. They are available in two different forms, i.e., either as filaments or pellets. The choice of the material form depends on the type of the printer. Filaments are used in the printers equipped with the filament spool holder and adjusted thermal nozzle, whereas pellets are utilized in the machines with the heated cartridge [76]. The most common materials available in the form of filament include acrylonitrile butadiene styrene, polycaprolactone (PCL), poly(lactic acid) (PLA), polyetheretherketone (PEEK), and polybutylene terephthalate [[77], [78], [79]]. Due to the production process, which requires at least one extra heating step that carries a risk of material's properties change or degradation, the number of inks available as filaments is limited. Moreover, printing with filaments does not allow easy employment of composites combining more than one type of material [80,81]. The use of pellets is more versatile. The typical materials available in pellet form are PCL, PLA, or poly(ester)urethane (PU) [82,83]. The material ink production process is simpler and less expensive. Using pellets facilitates printing of complex compositions, including multilateral inks and the addition of particles or molecules to improve the material printability or the properties of the final scaffolds [84].

Similar to FDM, the MEW approach utilizes thermoplastic polymers, typically with low T_m_. The well-printable materials are characterized by the high molecular weight that ensures enough entanglements, relatively high viscosity, and low conductivity to achieve stable and continuous jet formation [44]. The golden standard material for MEW is PCL; however, other polymers were also proposed, including poly(l-lactic acid) (PLLA), poly (ε-caprolactone)-poly (ethylene glycol) (PCEC), poly(propylene), or poly(vinylidene fluoride) [85,86]. A more detailed description of MEW polymers and their requirements can be found in an excellent recent review [44].

Finally, the NFES approach is mostly based on using polymers that are soluble in volatile solvents [87]. With NFES, natural and synthetic polymers can be processed, e.g., alginate, gelatin, PCL, or PLLA [54,[88], [89], [90], [91]]. The use of organic solvents (e.g., acetic acid, poly (ethylene oxide), or 1,1,1,3,3,3-hexafluoro-2-propanol (HFIP) [54,90,92]) restricts cell encapsulation and carries a risk of cytotoxicity. On the other hand, the printer set-ups are simpler as the heating elements are not needed. Moreover, uniform fibers thinner than in MEW printing can be obtained using high conductivity solvents [93,94].

The materials that can be processed using different EBP techniques (hydrogels, thermoplastics, and polymers soluble in volatile solvents) have their specific properties beneficial for successful tissue regeneration. Therefore, various studies employed a combination of those materials, and relevant printing approaches, to achieve synergistic effects. For example, a combination of hydrogel matrix with printed thermoplastic allows for encapsulation of cells in biocompatible hydrogel and the mechanical strengthening of the scaffold by the thermoplastic mesh [44,95].

## 3 Cues for cells

Using EBP approaches, various cues can be introduced to the scaffolds to direct cellular performance and tissue maturation. We systematically review them in the following chapters.

### 3.1 Physical cues for cells

Physical cues cover the whole spectrum of the substrate properties, excluding the addition of soluble factors or biochemical reagents [96]. The topography of the scaffold, such as pore size and shape, fiber thickness, fiber alignment, and degree of porosity, can direct cellular response. Scaffold architecture was shown to influence cell attachment, shape, proliferation, or migration patterns [22]; it is crucial for transporting nutrients and gas [97]. Mechanical properties, including substrate stability and stiffness, and electro-conductive properties, are other physical signals that influence cell performance [96]. Physical cues, especially those connected to the scaffold's design, can be relatively easily tuned with EBP methods.

#### 3.1.1 Pore size

One of the most commonly studied cell cues is pore size, tunable with EBP in a technique-dependent range. The smallest pore size reported for NEFS was 10 μm [61]; however, as the size of mammalian cells is at the level of tens of micrometers, the smaller pores would not be effective for cell guidance [54]. With MEW, a pore size of 40 μm [55] and bigger (up to hundreds of microns) can be obtained [[98], [99], [100]]. Typically, the reproducibility limit is reported at the level of 200 μm. The pores smaller than that are challenging to obtain with high reproducibility and accuracy [101] due to residual charges that cause the jet's instabilities, thus affecting the accuracy of fiber deposition [102]. For the FDM and extrusion bioprinting, 100 μm or bigger pores are typically obtained [103,104]. The smaller distance between the strands often leads to strand merging [33]. This section describes the influence of pore size on cellular performance in the scaffolds produced via different methods, starting with the approach allowing the smallest pore sizes.

##### 3.1.1.1 Near Field Electrospinning

Liang et al. [61] studied cell orientation on PCL micro-line arrays obtained via NEFS with interfiber distance ranging from 10 μm to 60 μm. The alignment of the NIH-3T3 cells was diminished with an increase in the fiber spacing [61]. Similar results were reported for NFES alginate fiber patterns, indicating higher alignment of HEK 293T cells while decreasing interfiber distance from 10 μm to 80 μm. For the spaces between fibers smaller than 40 μm, cell orientation was more strongly influenced by the nanofibers orientation, resulting in alignment of the majority of cells in the direction of the fibers in contrast to random cell alignment detected for higher interfiber distances [54]. For poly(γ-methyl l-glutamate) scaffolds, the NIH-3T3 cell coverage after four days increased from 66.42% to 88.38%, when the distance between fibers was increased from 250 μm to 750 μm, respectively, indicating slightly higher cell proliferation for scaffolds with bigger pores [88].

##### 3.1.1.2 Melt Electrowriting

The smallest reported square pores obtained with MEW ranged from 40 μm to 100 μm. They were designed to investigate the M2-like polarization of human macrophages (differentiation toward the M2-type), which is identified by their elongation. M2 macrophages play a role in regulation and wound healing and release anti-inflammatory cytokines. M1 macrophages are usually pro-inflammatory and, if present for an extended time, can cause foreign body response. As this can lead to a failing of scaffold integration, M2 differentiation is preferred. The results have shown that all scaffolds, irrespective of the pore size, promoted the elongation of human macrophages; however, the smallest pores (40 μm square mesh) led to the highest percentage of elongated cells and polarization toward M2 type. Furthermore, in the 40 μm scaffolds, the length of the elongated cells was the highest. For the pore size increase from 40 μm to 100 μm, the number of elongated cells decreased from 50% to 20%, respectively. M2 marker CD163 was upregulated for scaffolds with pore sizes smaller than 60 μm. The authors concluded that scaffolds with the smallest pore size of 40 μm lead to polarization of macrophages toward the M2 type [55].

In another study, Brennan et al. [98] fabricated PCL melt electrowritten scaffolds with 100 μm, 200 μm, and 300 μm square pores, fiber diameter 4.01 μm ± 0,06 μm, to investigate the impact of pore size on human bone marrow stem cells (hBMSCs). Proliferation, morphology, adhesion, osteogenesis, and mechanosignaling were studied. The scaffolds with the smallest pore size showed the greatest seeding efficiency. However, higher proliferation was observed for the bigger pores. The cells were elongated and stretched along the fibers in the scaffolds with 200 μm and 300 μm pore sizes, while cells spread across the pores for the smaller pore size. Moreover, 100 μm scaffolds have shown enhanced deposition of minerals and collagen, indicating the best osteogenic properties of these scaffolds [98]. PCL scaffolds with an interfiber distance of 100 μm, 200 μm, and 400 μm were also fabricated to reinforce a Matrigel matrix. The results showed that smaller pores hindered the proliferation of fibroblasts compared to bigger pores (200 μm and 400 μm) [105]. In contrast, Xie et al. [99] have proved that higher human umbilical vein endothelial cells (HUVECs) proliferation rates were observed on the PCL scaffolds with smaller square pores (100 μm × 100 μm) in comparison to scaffolds with higher pore sizes (100 μm × 200 μm and 200 μm × 200 μm). The cells could fill the smallest pores after one week, while intermediate growth and some bridging were observed in the medium pore sizes. The scaffolds with the biggest pore size revealed the lowest cell growth rate and no filling of the pores. In addition, the study showed that the cells of different sizes displayed different morphologies. In a 200 μm square scaffold, smaller HUVECs (size below 100 μm) grew along the fibers and formed circles around the pores, whereas bigger bone marrow stem cells (BMSCs) (size over 200 μm) tended to bridge the pores [99]. Another study with PCL scaffolds further proved that the time needed for cells (osteoblasts) to bridge pores increases with the pore size. For 200 μm pore size, the cells bridged the pores after 14 days, while for 600 μm, the pores were not completely covered with cells even after 28 days. The authors have detected the linearity between pore size and the time needed for cells to bridge the pores [100].

Nguyen et al. [106] fabricated MEW PCL scaffolds with a pore size of 100 μm, 200 μm, and 300 μm to examine the effect of pore size and fiber sagging on the attachment and growth of fibroblasts (NIH-3T3). While the pore size of scaffolds increases, the MEW fibers start to hang and consequently sag. The 100 μm scaffold showed no sagging, while intermediate and most pronounced sagging were observed for the 200 μm and 300 μm pore scaffolds, respectively. One day after seeding, the 100 μm scaffold showed the highest fibroblast attachment to all scaffold layers. The 300 μm scaffolds had the lowest number of cell attachments. However, increased numbers of attached cells at the intersection of the printed fiber were detected. The fibroblast attachment was observed on sagged fibers only to the freely available top surface. Two weeks after seeding, the 100 μm and 200 μm pores were entirely filled with cells, while the 300 μm pores were filled up to 31.78% [106].

##### 3.1.1.3 Fused Deposition Modeling

Compared to the NFES and MEW methods, FDM offers reproducible pores in higher sizes (100 μm or bigger). Greamre et al. [107] have fabricated PLA scaffolds with square pores varying in size (150 μm, 200 μm, 250 μm) for bone regeneration. After three- and seven-day cell culture, it was observed that hBMSCs were spread over the mesh and began to close the pores. However, the cells could not close any of the pores after seven days. The cells have shown homogenous distribution throughout the scaffold regardless of the pore size [107]. In a similar approach, PEEK scaffolds with square pores (300 μm, 450 μm, and 600 μm) were fabricated for bone substitution. The cell seeding efficiency of hBMSCs was the highest for the 300 μm scaffolds and decreased with increasing pore sizes. Even though scaffolds with 300 μm pore size have the largest surface area favoring cell adhesion, the cell viability on those scaffolds was lower than on the rest of the scaffolds. This may be due to the fact that the larger pores allow for more efficient transport of oxygen and nutrients, which are essential for cell growth. After seven days, the expression of osteogenesis genes was higher on the sample without any pores (control sample). However, after 14 days, the gene expression was higher for porous scaffolds, the most pronounced for 300 μm and 450 μm pore sizes. Moreover, *in vivo* studies have shown that 450 μm scaffolds had increased blood perfusion 12 weeks after implantation. At 4 and 12 weeks post-implantation, more enhanced bone formation was observed for 450 μm scaffolds than for the 300 μm and 600 μm scaffolds. The authors concluded that the scaffolds with 450 μm pores were most suitable for bone substitution due to good proliferation and osteogenic differentiation. Furthermore, these scaffolds were favorable for bone ingrowth and vascular perfusion [108].

In another study, PLA-epoxy scaffolds with dual porosity were fabricated using gas foaming and 3D printing. The scaffolds were produced with sub-macro (10 μm – 60 μm) and macro-sized (200 μm – 300 μm) pores. The results showed that the proliferation rate of NIH-3T3 cells on the neat PLA scaffolds (only macro-sized pores) was five times lower than on the scaffolds with dual porosity. Moreover, the cell attachment on neat scaffolds was very low compared to those with dual porosity. In conclusion, dual pore scaffolds have a higher surface area needed for cell attachment, affecting cell proliferation [109].

##### 3.1.1.4 Extrusion bioprinting

Using extrusion bioprinting, Sadeghianmaryan et al. [68] have fabricated chitosan scaffolds with pore sizes ranging from 2 to 4 mm for cartilage tissue. The attachment of chondrocytes seeded on the scaffold increased with the decrease in the pore size because of the higher available surface area. Most of the cells after 24 h of culture had characteristic round morphology typical for these cells [68]. In another study, the gelatin-based square-mesh scaffolds with 200 μm, 302 μm, and 382 μm pores were fabricated to determine the relationship between pore size and gene expression of mesenchymal stem cells (MSCs). The data indicate that cells seeded post-printing on the scaffolds with smaller pores (200 μm) formed a quasi-2D layer around the scaffolds preventing infiltration of the scaffolds. In the case of the biggest pores (382 μm), the cells were attached to the strands and did not bridge the pores. The medium pores (302 μm) forced cells to aggregate between parallel strands. Furthermore, MSCs with HUVECs seeding on the 302 μm pore size scaffolds have enhanced angiogenic paracrine activity (secretion and expression of angiogenic factors) and cell spreading [110]. Gelatin scaffolds with a different square-shaped pore size (435 μm–∼800 μm) were also fabricated to examine their effect on the cellular behavior of seeded dermal fibroblasts (HDFs). HDFs in the 3D gelatin scaffolds with pore size bigger than 580 μm proliferate faster than in the scaffold with pore size 435 μm after 14 days of culture [66].

##### 3.1.1.5 Summary

The pore size was mainly used to instigate cell attachment, alignment, and proliferation. The data show that the smaller pores allow for better seeding efficiency while bigger pores provide a higher proliferation rate due to more efficient transport of nutrients and oxygen. Moreover, the cells tend to spread across the small pore sizes (100 μm and less) while the elongated cells along the fibers can be observed for scaffolds with bigger pores. In the scaffolds composed of parallel fibers only, the cells showed clear alignment in the direction of fibers if the distance between parallel fibers was in the cell size range or smaller. If the distance between fibers was bigger, the random cell arrangement was more commonly observed. The most commonly used methods to produce scaffolds with different pore sizes are MEW and FDM. However, it should be noted that MEW allows creating scaffolds with a broader range of pore sizes than FDM due to the resolution limit of the latter one. A summary of the studies employing pore size as a cue is presented in Table 1.

**Table 1:** Pore size and its influence on cell behavior.

Printing method	Tissue	Cell cue	Main material(s)	Cell type(s)	Cell response	Ref.
**NFES**	Not specific	Micro-line arrays (10 μm–60 μm interfiber distance)	PCL	NIH-3T3	Higher cell alignment with decreasing interfiber distance.	[61]
		Increasing interfiber distance (10 μm–80 μm)	Alginate	HEK 293T	Higher cell alignment with decreasing interfiber distance.	[54]
		Increasing interfiber distance (250 μm–750 μm)	PCL	NIH-3T3	Higher cell proliferation for scaffolds with bigger pores.	[88]
**MEW**	Bone	Square pore size (100 μm, 200 μm, 300 μm)	PCL	hBMSCs	Higher cell seeding efficiency, enhanced mineral and collagen deposition for the smallest pores.	[98]
	Skin	Pore size (100 μm × 100 μm, 100 μm × 200 μm and 200 μm × 200 μm)	PCL	HUVECs	Highest cell proliferation rate on the smallest pore size.	[99]
	Not specific	Square pore size (100 μm, 200 μm, 300 μm)	PCL	NIH-3T3	Improved cell attachment to the smallest pore scaffolds.	[106]
		Square pore size (100 μm, 200 μm, 400 μm)	PCL/Matrigel	Fibroblasts	Enhanced proliferation rate for bigger pores (>100 μm).	[105]
		Square pore size (40 μm–100 μm)	PCL	Human macrophages	The decreased number of elongated cells with increasing pore size.	[55]
		Square pore size (200 μm–600 μm)	PCL	Osteoblasts	Increased time for pore bridging with increased pore size.	[100]
**FDM**	Bone	Square pore size (150 μm, 200 μm, 250 μm)	PLA	hBMSCs	Homogeneous cell distribution regardless of the pore size.	[107]
		Square pore size (300 μm, 450 μm, 600 μm)	PEEK	hBMSCs	Higher bone formation *in vivo* for scaffolds with pore size 450 μm.	[108]
	Not specific	Sub-macro (10 μm–60 μm) and macro-sized (200 μm–300 μm) pores	PLA	NIH-3T3	Higher proliferation rate on scaffolds with dual porosity.	[109]
**Extrusion printing**	Cartilage	Square pore size (2 mm–4 mm)	Chitosan	Chondrocytes	Higher cell attachment on smaller pore size scaffolds with round morphology.	[68]
	Blood vessel	Square pore size (200 μm, 302 μm, and 382 μm)	Methacrylamide-modified gelatin	MSCs and HUVECs	Pore bridging for the smallest pore sizes. The angiogenic activity of HUVECS on bigger pores.	[110]
	Not specific	Square pore size (435 μm–∼800 μm)	Gelatin	HDFs	Higher proliferation rate on scaffolds with bigger pore sizes (>435 μm).	[66]

#### 3.1.2 Pore shape and fiber alignment

Scaffolds with various pore shapes (e.g. triangle, square, hexagon, sinusoidal, diamond) were printed [53,101]. The most commonly used pore shape is a square mesh [88,108]; however, the EBP allows for high flexibility in the designs, including well-organized and random alignment of the fibers. Importantly, in many studies, the design of the pore shape is interwoven with pore sizes.

##### 3.1.2.1 Near Field Electrospinning

With the use of NFES, GelMA was reinforced with a 100 μm square pore size poly (ε-caprolactone)-poly (ethylene glycol) (PECL) scaffold to form a 3D fiber-reinforced hydrogel for cornea treatment. The reinforced GelMA scaffolds inhibit fibroblasts' differentiation of limbal stromal stem cells in serum-containing media, not observed in pure hydrogel scaffolds. Moreover, the results have shown that the cells in reinforced hydrogel scaffolds were elongated along PECL fibers [89]. In another study, Fattahi et al. [111] analyzed the effect of anisotropic properties of electrospun poly(methyl methacrylate) (PMMA) fibers reinforcing collagen gels, with embedded hMSCs, on cellular behavior (cell migration and proliferation). The cells tended to remodel and extend along the direction of the fibers inside the gel. In contrast, cells in the absence of PMMA fibers (embedded in pure hydrogel) have nearly uniform actin cytoskeleton's orientation in all directions [111].

Gill et al. [90] have fabricated gelatin scaffolds with different laydown angles (0°, 30°, 45°, and 90°). The laydown angle is the angle between fibers in consecutive printed layers. Human glioblastoma cells were seeded in the form of aggregates after printing. The results have shown higher initial cell attachment on all designs, except 0° laydown angle, due to the higher surface area formed by the stacked fibers. The cells migrated from the seeded aggregates, and their migration was guided by fiber topography (i.e. the cells were migrating along the fibers) [90].

##### 3.1.2.2 Melt Electrowriting

**Fig. 3:**
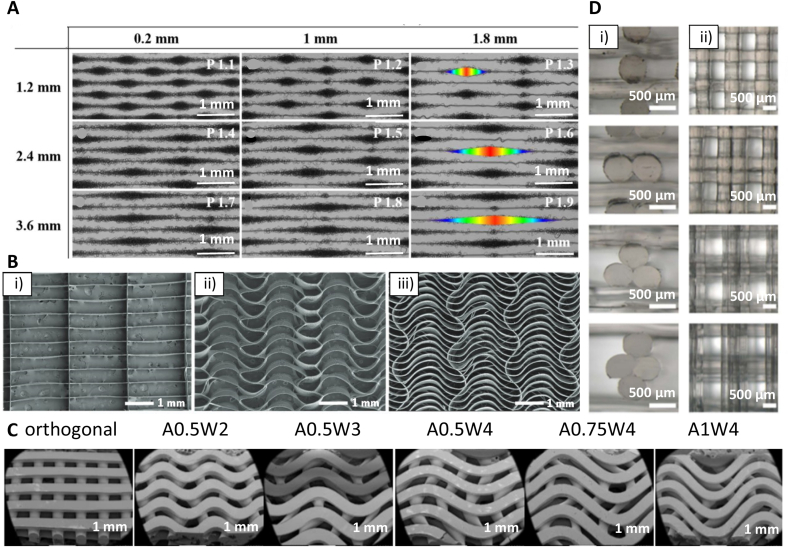
Examples of different pore shapes and fiber alignments in produced scaffolds. (A) SEM images of coil compacted PCL scaffolds with various interfiber distances and periodic diagonal lengths controlled by changing printing speed. (B) SEM images of PCL MEW scaffolds with 20 layers: i) rectangular pore shape (0.5 mm × 2 mm), and serpentine scaffold with 2 mm radial pore size with ii) 0.5 mm, and iii) 0.25 mm circumferential pore size. (C) SEM images of FDM scaffolds with linear and sinusoidal fiber alignment. **A** corresponds to interfiber distance, **W** corresponds to a wavelength of sinusoidal fibers. (D) PLA FDM scaffolds with different fiber cross-sections: circular as control, bilobed, trilobed, quadrilobed (from top to bottom), i) cross-section of printed scaffolds, ii) top view of printed scaffolds. (A) Adapted under the terms of the Creative Commons Attribution 4.0 International License from Ref. [113], Copyright © 2020 Springer Nature. (B) Adapted with permission from Ref. [117], Copyright © 2020 Wiley-VCH. (C) Adapted under the terms of the Creative Commons Attribution License from Ref. [119], Copyright © 2020 MDPI. (D) Adapted with permission from Ref. [123], Copyright © 2021 IOP Publishing Ltd.

MEW was used by Gwiazda et al. [112] to fabricate PCL scaffolds for bone-ligament-bone regeneration constructs. The authors created scaffolds with aligned, crimped, and random patterns. hMSCs were seeded on the scaffolds post-printing, and the influence of the substrate design was analyzed. It was shown that the cells initially grow along the fibers and at day 1 of cell culture reveal clear directionality, following the arrangement of the fibers. One week after seeding, the topological guidance was still effective in the aligned and crimped scaffolds. However, the directed cell orientation was maintained only in the aligned scaffold after two to three weeks of *in vitro* expansion. The cell directionality disappeared in the crimped and random scaffolds after the cells reached confluence. It was also shown that the *in vitro* culturing led to softening of the scaffold/cell construct with crimped and aligned designs, resulting in a significantly reduced slope of the stress-strain curves of these scaffolds. Random designs were unaffected. However, the pore design did not influence cell metabolic activity, neither proliferation rate, nor differentiation toward osteoblast lineage [112]. In another study, hierarchically ordered 3D coil compacted PCL scaffolds have been fabricated with different densities of coils (Fig. 3A). The coil density could be altered by changing the collector plate speed; lower speed led to a higher density. After seven days of cell culture, the printed scaffold showed significantly increased cell proliferation than controls cultured on tissue culture plastic. The scaffolds with more dense coils have a higher surface area and displayed enhanced cell-cell interactions and cell-extracellular matrix interactions compared to less dense designs [113].

Paxton et al. [114] have fabricated PCL tubular aligned (fibers in consecutive layers printed precisely on top of each other, 0.5 mm^2^ pore size) and non-aligned scaffolds (inconsistent fiber placement in consecutive layers, varying pore size: 0.02 mm^2^ – 0.33 mm^2^) with 20°, 50°, and 90° laydowns angles. Murine calvarial pre-osteoblast cells (MC3T3-E1) were seeded on top of the scaffolds. The scaffolds with the lower laydown angles (20° and 50°) showed more uncontrolled cell bridging (cells crossing the scaffolds in different directions) in comparison with the 90° scaffolds, where the cells first have grown on the fibers and then started to close pores. In the non-aligned scaffolds, cells bridge and fill areas with smaller pores and gradually expand to bigger pores. However, after 21 days, predictable and consistent growth and pore bridging have also been observed in the aligned scaffolds. In these scaffolds, the cells followed the direction of the PCL fibers more compared to the non-aligned scaffolds [114]. Tubular PCL scaffolds with different structures (square and rhombus with 200 μm pore size and random) were also fabricated for kidney tissue engineering. Human conditionally immortalized proximal tubular epithelial cells (ciPTECs), and HUVECs were seeded on separate scaffolds. Cell-specific behavior was observed. HUVECs could not bridge the pores, while the ciPTECs were able to form tight monolayers. The rhombus scaffolds enabled unidirectional cell orientation in the scaffold's fibers direction, which is not observed for square and random designs. Furthermore, rhombus scaffolds led to an increased collagen type IV deposition. The proximal tubule in the kidney is important for secretion and, consequently, functional transporters are essential. An increase in the gene expression of the transporters organic cation transporter 2 (OCT2) P-glycoprotein was observed in the rhombus scaffolds. The authors expect that the aligned cytoskeleton caused the increased transporter gene expression in those scaffolds [115].

Tourlomousis et al. [116] used machine learning metrology to investigate cell confinement in MEW scaffolds. The cell morphology of neonatal human dermal fibroblasts (NHDFs) was investigated in PCL scaffolds with varying designs. Scaffolds of 200 μm pore size and laydown angle of either 90^o^ or 45° were prepared. Additionally, scaffolds with randomly oriented fibers were created with solution electrospinning (SES). Different electrospinning times (1 min and 3 min) were applied to produce SES scaffolds with varying fiber coverage of the glass substrate. The results showed that cells cultured on the 1 min SES scaffolds (non-uniform fiber coverage) showed a considerable variance in the cell area, which can be explained by the heterogeneity in the topography of these scaffolds. Furthermore, the NHDFs on these scaffolds developed lamellar shapes and were widespread. The cells on the 3 min SES scaffolds (uniform fiber coverage) had the smallest cell area. The MEW designs showed no statistical difference in the mean cell area. NDHFs seeded on the 90° MEW scaffolds were mainly attached along fibers and at crossing points of fibers while seeded on the 45° MEW scaffolds developed into triangular lamellar shapes [116].

In another study, MEW was utilized to create PCL scaffolds for heart valve tissue engineering. 20-layered serpentine scaffolds (radial pore size of 2 mm and circumferential pore size of 0.25 mm or 0.5 mm) and scaffolds with straight fibers (0.5 mm x 2 mm pore size) were created (Fig. 3B). Cell's pore bridging was observed one week after seeding of human umbilical cord vein smooth muscle cells (hUVSMCs) in the serpentine scaffolds but not in the straight fibers scaffolds. This can be explained by the higher surface area for the cell attachment in the design containing curvy fibers. However, after two weeks, pores in all scaffolds were entirely filled. In the next step, the MEW scaffolds were embedded in a fibrin gel loaded with hUVSMCs. Collagen I and III deposition was observed in the hybrid construct. A fibrin-only control was heavily contracted after one week of culture. In contrast, a hybrid construct maintained shape stability after two weeks of culture due to mechanical support provided by the MEW scaffold. The lack of shrinkage is essential for the sufficient closure of tissue-engineered heart valves [117]. Finally, Castilho et al. [118] fabricated a blend of poly(hydroxymethylglycolide-*co*-ε-caprolactone) (pHMGCL) with PCL (weight ratio 40:60) scaffolds for cardiac tissue engineering to better mimic the mechanical environment and structural organization of cardiac tissue. An enhanced alignment of cardiac progenitor cells (CPCs) was observed in rectangular pHMGCL/PCL scaffolds, whereas random cell arrangement was observed in square scaffolds [118].

##### 3.1.2.3 Fused Deposition Modeling

Ji et al. [119] fabricated PCL scaffolds for bone tissue engineering with linear and sinusoidal fiber alignment (Fig. 3C) using the FDM technique. The results have shown that seeded hMSCs elongate along with the wavy designs and take the shape of curved strands, while on the linear pattern, the shape of the cells was more rounded. Moreover, scaffolds with the sinusoidal pattern have shown enhanced differentiation into bone cells than those with the linear design. According to the authors, higher osteogenesis for wavy scaffolds was the effect of the curvature, which caused the alignment and stretching of the cells. As a result, the cells developed mature focal adhesions, which promote osteogenesis [119]. PCL was also printed with 60 μm strand diameter into square shape grids. The printed stands had internal micro- (1 μm – 2.4 μm) and nano- (200 nm – 1000 nm) porosity. The authors have used for printing the bubbled viscous PCL/dichloromethane (DCM) solution, inducing additional porosity after printing due to DCM evaporation. The strands without internal porosity (nonporous) have been used as control. Mesenchymal stem cells (MSCs) were seeded post-printing. Quantification of collagen II production at day 28 of cell culture in the chondrogenic differentiation medium without TGF-β1 showed the chondrogenic differentiation enhancement by the porous topography as evidenced by increased chondrogenic markers (sGAG and collagen II production). In osteogenic induction medium with dexamethasone, MSCs on porous scaffolds exhibited improved osteogenic differentiation as indicated by the enhanced enzymatic activity of alkaline phosphatase (ALP) at day 14 and considerably higher production of osteocalcin at day 28 compared to the nonporous scaffolds. These results suggest an inherent effect of architecture and topography for directing stem cell differentiation [120]. In another study, PU scaffolds with a pore size of 500 μm and laydown angles of 90° and 60° have been printed to investigate the differentiation of BMSCs into chondrocytes. The characteristic ECM components for cartilage (GAGs, Col1) were observed for both designs. However, the 60° pattern has shown higher content of GAGs and collagen in comparison to the 90° pattern, indicating that the first one can favor chondrogenic differentiation. Moreover, the cell infiltration of the scaffold was higher on a 60° pattern. The upregulation of chondrogenic markers (Col2, Sox5, Sox6, Sox9) was observed for both types of scaffolds after 14 and 28 days of cell culture. The authors concluded that the 60° pattern scaffolds promote chondrogenic differentiation due to the lower surface area, allowing more cell-cell interactions and reducing the focal adhesion needed for chondrogenesis [121]. Theodoridis et al. [122] have studied the effects of pore geometry of 3D-printed PCL scaffolds on chondrogenic differentiation of adipose tissue-derived mesenchymal stem cells (ADMSCs). Scaffolds with a rectangular layered pattern with an average pore size of 200 μm, a triangular pattern with an average pore size of 210 μm, and a 3D honeycomb-like pattern with a hexagonal and small rectangular shapes with an average pore size of 425 μm have been fabricated. It was observed that a triangular pattern design was the most beneficial for chondrogenic differentiation as indicated by elevated expression levels of chondrogenic markers SOX9 and ACAN. However, after 26 days of culture, cells seeded on 3D honeycomb-like pattern scaffolds revealed higher proliferation, infiltration, and migration throughout the entire construct; those scaffolds were also characterized by seven-fold increased mechanical properties compared to the scaffolds before the cell culture [122].

Interestingly, Mainardi et al. [123] investigated cellular response to the fibers' shape. They used PLA scaffolds (diameter of 8 mm and a height of 4 mm) with four different geometries, three so-called multilobed designs: bilobed, trilobed, quadrilobed, and one circular design as control (Fig. 3D). A scaffold was placed in an oscillating platform to perform the dynamic cell seeding of the MG63 osteoblasts, fibroblasts, and chondrocytes. The medium with the cells was flowing through the scaffolds due to the effect of the bidirectional oscillation of the chamber. The results have shown that seeding efficiency for every type of cell was higher for multilobed scaffolds than circular ones, with 3.61-fold higher efficiency for trilobed scaffolds. The increased cell attachment on trilobed scaffolds was attributed to the asymmetrical shape of the fibers in the flow direction, causing fluid dynamics alteration. Consequently, the path followed by cells was changed compared to the circular scaffolds enhancing the cell seeding by providing more suited sites for cell adhesion. In addition, cells were more likely to spread parallel to the fiber axis in multilobed scaffolds while having a rounded shape for circular scaffolds due to the niches between two parallel fibers, which were not present for the circular scaffolds [123].

##### 3.1.2.4 Extrusion bioprinting

Tijore et al. [62] 3D printed parallel strands from gelatin on gelatin hydrogel film crosslinked with the microbial transglutaminase, with the effecting spacing of ∼250 μm – 300 μm or 800 μm between two adjacent microchannels. hMSCs were seeded on the top of the strands and on a uniform gelatin film as a control. The cardiomyocyte markers β-MHC and cardiac troponin T were highly expressed within the cells seeded on the strands but not on the gelatin film. Moreover, cardiomyocyte beating potential was evaluated with neonatal rat cardiomyocytes (CMs), and remarkable consistent rhythmic beating was revealed for the cells seeded on the strands compared to controls. The authors assigned this effect to a more organized sarcomere structure developed in aligned CMs growing on the microchannels [62].

##### 3.1.2.5 Summary

The results imply that the pore shape can significantly influence cell behavior. Especially MEW, which allows depositing fibers in the dimensions close to the single-cell size, emerged recently as a powerful approach to steer cellular response via the pore shape. Yet, obtaining volumetric scaffolds with this approach is still a challenge, and for more extensive constructs, the use of FDM or extrusion printing is more feasible. A summary of the studies employing pore shape as a cue is presented in Table 2.

**Table 2:** Pore shape and its influence on cell behavior.

Printing method	Tissue	Cell cue	Main material(s)	Cell type(s)	Cell response	Ref.
**NFES**	Cornea	Fiber guidance	PECL, GelMA	Limbal stromal stem cells	Restricted fibroblasts' differentiation on reinforced hydrogel scaffolds, cells elongated along the fibers.	[89]
	Not specific	Fiber guidance	PMMA/collagen	hMSCs	Cells elongated along the fibers reinforcing hydrogel.	[111]
		Various laydown angles (0°, 30°, 45°, and 90°)	Gelatin	Human glioblastoma cells	Enhanced migration and proliferation on higher laydown angles.	[90]
**MEW**	Bone	Aligned, crimped, and random patterns	PCL	hMSCs	Maintained cell orientation on aligned scaffolds after 21 days of cell culture.	[112]
		Fiber alignment (compacted coils)	PCL	hMSCs	Increased cell proliferation and cell-cell interactions on more dense coils.	[113]
		Various laydown angles (20°, 50°, and 90°)	PCL	MC3T3-E1	Enhanced directionality of cells along the PCL fibers for higher laydown angle.	[114]
	Cardiac	Radial, circumferential and rectangular pore shape	PCL	hUVSMCs	Faster pore bridging for the radial pattern.	[117]
		Rectangular and square pore shape	pHMGCL/PCL	CPCs	Enhanced alignment of cells for rectangular scaffolds.	[118]
	Kidney	Tubular scaffolds (square, rhombus, and random pore shape)	PCL	ciPTECs, HUVECs	Unidirectional cell alignment for rhombus pores with increased gene expressions.	[115]
	Not specific	Various laydown angles (45° and 90°)	PCL	NHDFs	Elongation along the fibers for higher laydown angle while the lamellar shape of cells on the smaller laydown angle.	[116]
**FDM**	Bone	Linear and sinusoidal fiber alignment	PCL	hMSCs	Enhanced osteogenic differentiation on sinusoidal scaffolds.	[119]
		Micro (1 μm–2.4 μm) and nano (200 nm–1000 nm) porosity of the deposited strands	PCL	MSCs	Improved osteogenic and chondrogenic differentiation in osteogenic and chondrogenic induction medium, respectively, for scaffolds composed of fibers with micro- and nanoporosity.	[120]
	Cartilage	Various laydown angles (60° and 90°)	PU	BMSCs	Enhanced chondrogenesis on rhombus scaffolds.	[121]
		Rectangular, triangular, honeycomb pore shapes	PCL	ADMSCs	Enhanced chondrogenic differentiation on triangular scaffolds.	[122]
	Not specific	Different geometry of fiber cross-section	PLA	MG63, fibroblasts, chondrocytes	Higher seeding density for multilobed scaffolds.	[123]
**Extrusion printing**	Cardiac	Microchannels with different interfiber distances	Gelatin	hMSCs, CMs	Upregulated cardiomyocytes marker levels, cardiomyocytes rhythmic beating with CMs regardless of interfiber distance.	[62]

#### 3.1.3 Fiber thickness

Fiber thickness can be typically easily tuned by changing the printing parameters (e.g. pressure, voltage, nozzle, printing speed, depending on the specific technique) or printing approach. The smallest fiber diameter on the nano-level can be obtained using NFES, while MEW allows printing the fibers in the range of a few micrometers [48,49]. The thickest fibers can be obtained using FDM or extrusion bioprinting (in the range of hundreds of μm) [124].

An interesting study to analyze the influence of the fiber size on cell performance was proposed by Xie et al. [99]. The authors used MEW to print PCL scaffolds with varying fiber diameters (3 μm – 22 μm) and analyzed the behavior of two cell types with different sizes, namely BMSCs (typically cells with 200 μm in length) and HUVECs (less than 100 μm in length). The BMSCs adhered to thick fibers (printed at 500 mm/min), whereas they wrapped around thin fibers (printed at 2000 mm/min). In the scaffolds consisting of only thick fibers, BMSCs filled the pores by growing around them and systematically closing the space from the edges to the center. If a pore consisted of thick fiber in one direction and thin fibers in the other, the BMSCs exhibited oriented growth, bridging the pore from thin fiber to opposite thin fiber. The cells on scaffolds consisting of thin fibers only showed random growth, with cells bridging opposite or neighboring fibers. HUVECs were not affected by the fiber thickness and revealed only circular growth around the pores. This effect was assigned to the fact that HUVECs are smaller than BMSCs and do not have enough length to bridge the pores of the printed samples, and could only adhere and grow on single fibers [99].

**Fig. 4:**
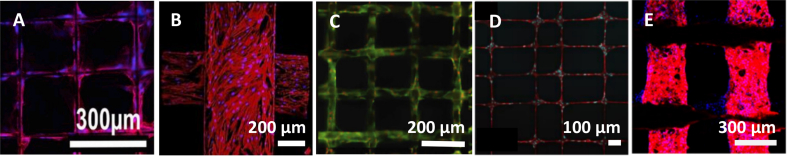
Cell cultured on the PCL scaffolds with different fiber diameters. hMSCs cells one week after seeding on: (A) MEW scaffold, (B) FDM scaffold. MC3T3-E1 cells seeded on: (C) MEW scaffold after four days, (D) NFES scaffold after two days, (E) FDM scaffold after one week. (A) Adapted with permission from Ref. [112], Copyright © 2020 Elsevier. (B) Adapted under the terms of the Creative Commons Attribution License from Ref. [119], Copyright © 2020 MDPI. (C) Adapted with permission from Ref. [100], Copyright © 2020 Elsevier. (D) Adapted with permission from Ref. [111], Copyright © 2017 Wiley-VCH. (E) Adapted with permission from Ref. [125], Copyright © 2014 American Chemical Society.

PCL square mesh (200 μm × 200 μm) scaffolds with a fiber diameter of around 20 μm and 530 μm were printed using MEW [112] and FDM [119], respectively. The hMSCs seeded on MEW scaffolds grew around the MEW fibers and started closing the pores after one week of cell culture (Fig. 4A). On the contrary, cells on FDM scaffolds have only grown along the fibers, and after one week of culturing, they coved the whole surface of fibers but did not fill in the pores (Fig. 4B) [112,119]. Also, the performance of MC3T3-E1 cells on fibers with different diameters can be observed when comparing scaffolds prepared with NFES [111], MEW [100], and FDM [125] (Fig. 4C–E). After two days of culture, the cells on the 2 μm diameter fibers obtained via NFES, were well attached and followed the fiber orientation. The cells did not grow between the fibers due to the pore sizes exceeding 100 μm. If the interfiber distance was lower than 100 μm, the cells grew between fibers [111]. For MEW PCL scaffolds with fiber diameters of around 50 μm low attachment of cells was noticed during the initial seeding time; however, after four days, the cells started proliferating on fibers and slowly closing pores for scaffolds with a pore size of 200 μm. The results indicate that pores' closing begins at the place with high curvature (corners of pores) and surface area [100]. The biggest fiber diameter (275 μm ± 28 μm) was measured for PCL scaffolds with a pore size of 746 μm ± 71 μm, fabricated using FDM. After one day, only a few cells were attached to the surface of fibers, but the number grew over time, and after seven days, the whole surface of fibers was covered with cells. The data suggest that the cells spread along the thick fibers rather than (or before) bridging the pores, possibly due to the high surface area provided by the fibers [125].

##### 3.1.3.1 Summary

MEW approach is the most flexible in adjusting fiber thickness, as a broad range of fiber sizes can be obtained without changes in the hardware (the same nozzle can be used) solely via tuning printing parameters. In turn, extrusion printing of hydrogels is the most challenging in optimizing fiber diameter, as it typically requires taking into account multiple rheological characteristics (such as crosslinking kinetics, relaxation time, viscosity). There are also studies introducing gradient scaffolds with different fiber thicknesses in one design, where the nanometer fiber mats were employed as a catching layer which enhanced the seeding efficiency [126]. A summary of the studies employing fiber thickness as a cue is presented in Table 3.

**Table 3:** Fiber thickness and its influence on cell behavior.

Printing method	Tissue	Cell cue	Main material(s)	Cell type(s)	Cell response	Ref.
**NFES**	Not specific	Fiber thickness (∼ 2 μm)	PCL	MC3T3-E1	The fibers entirely covered by cells on day two of cell culture.	[111]
**MEW**	Bone	Fiber thickness (20 μm)	PCL	hMSCs	Cell spreading on fibers and after two weeks of culture bridging the pores.	[112]
	Not specific	Fiber thickness (3 μm–22 μm)	PCL	BMSCs, HUVECs	Adhesion of BMSCs to thick fibers and wrapping around thin fibers. Circular growth of HUVECs regardless of the fiber thickness.	[99]
		Fiber thickness (∼ 50 μm)	PCL	MC3T3-E1	Completely closed pores after two weeks of cell culture.	[100]
**FDM**	Bone	Fiber thickness (20 μm)	PCL	hMSCs	Spreading of cells in the direction of fibers.	[119]
		Fiber thickness (∼ 275 μm)	PCL	MC3T3-E1	Completely covered scaffolds by cells after one-week culture.	[125]

#### 3.1.4 Mechanical cues

Cells can sense and respond to physical forces and the stiffness of the substrate through focal adhesions. By tuning the local mechanical properties of the scaffold, a particular cellular response can be evoked [127]. Scaffold stiffness can influence cell size, shape, alignment and guide stem cells' differentiation into particular lineages [128]. A static or dynamic force applied to the scaffold can influence cell morphology, spreading, proliferation, or differentiation [129].

##### 3.1.4.1 Melt Electrowriting

MEW was used to produce PCL scaffolds with a hexagonal structure for human myocardial tissue engineering. The human-induced pluripotent stem cell-derived cardiomyocytes (iPSC-CM) were encapsulated in a collagen-based hydrogel and seeded on the printed scaffold. The pure PCL, 500 μm × 1000 μm rectangular scaffold, was used as a control. After seven days of culture, the cells covered both scaffold types and started to contract simultaneously across the whole scaffolds. However, the beating rate was higher for hexagonal scaffolds than rectangular ones. The authors assigned this effect to the fact that the hexagonal scaffolds had a higher elastic region (≈20–40 times) compared to the rectangular scaffolds. Moreover, hexagonal scaffolds have shown increased expression of maturation-related cardiac markers after 14 days of cell culture, which was not observed for rectangular scaffolds. The results indicate that the mechanical properties of the scaffolds influence cell contractions and cell maturation [43].

In another study, Castilho et al. [130] produced bi-layer scaffolds, composed of PCL mesh produced with MEW and GelMA casting, to mimic articular cartilage and examine the chondrogenesis under dynamic compression of scaffolds. The superficial tangential zone consisted of a densely distributed crossed fiber mat. A uniform box structure was employed in the middle and deep zones. The constructs were tested in cell culture without and with mechanical stimulation (a dynamic load ranging from 0 to ∼15%/20% amplitude strain, applied in a sinusoidal waveform with a frequency of 1 Hz). Production of glycosaminoglycan (GAG) and collagen II was observed. GAGs were deposited homogeneously in the whole scaffold, whereas collagen II was mostly found around cells. The results showed that the gradient constructs enhance cartilage formation when mechanically stimulated [130].

##### 3.1.4.2 Fused Deposition Modeling

In another study, Chae et al. [83] produced PU/PCL hybrid scaffolds with encapsulated hBMSCs for tendon replacement. The square scaffolds were subjected to static tension by fixing the scaffolds in clamps and applying constant tension. The uniaxial alignment of cells (along the longitudinal axis) under static tension was observed while cells on the scaffolds without tension were randomly aligned. This effect was desirable since the cells in the native tendon are arranged along the collagen fibers [83].

##### 3.1.4.3 Extrusion bioprinting

The stiffness of the hydrogel-based printed materials can be relatively easily tuned by changing e.g. crosslinking density, crosslinking chemistry, or bioink composition. The influence of scaffold stiffness on osteogenic differentiation and bone-like tissue formation was investigated using the bioprinting technique by Zhang et al. [67]. Two concentrations of alginate (0.8% w/v or 1.8% w/v) and gelatin (4.1% w/v) were mixed to fabricate soft and stiff scaffolds, respectively, with hMSCs encapsulated. The results demonstrated higher cell proliferation, enhanced ALP, and increased osteogenic differentiation in softer scaffolds than in stiffer ones. On day 42, significantly higher mineralization was observed in softer scaffolds. Immunohistochemistry staining revealed more osteocalcin protein expression in high mineral than low mineral regions [67]. The same authors in another study investigated the effect of alginate concentration on alginate/gelatin composite scaffold mechanical stiffness and the impact of the stiffness on MSCs' cell viability and morphology. With increasing content of alginate from 0.8% to 2.3%, the scaffold stiffness increased from 1.5 kPa to 14.2 kPa, respectively. After 14 days of cell culture, the cells formed a 3D interconnected network and exhibited a more spread morphology in the softer constructs. The reason was the lower diffusion rate of nutritions to the cells in stiffer constructs. The study proved that the alginate and gelatin composite scaffolds with lower stiffness (1.5 kPa) showed better cell spreading and migration [131]. In a similar study, the authors bioprinted scaffolds with stiffness variances using 2%, 5%, and 10% alginate solutions containing encapsulated mouse fibroblast cells (L929). The cell morphology, proliferation, and migration were investigated. On day 14 of cell culture, cell migration and proliferation throughout 2% and 5% alginate scaffolds were observed. However, in 5% alginate scaffolds cell aggregation was also detected. Cells formed spheroids in 10% alginate scaffolds due to the high network stiffness restricting cell mobility [132].

**Fig. 5:**
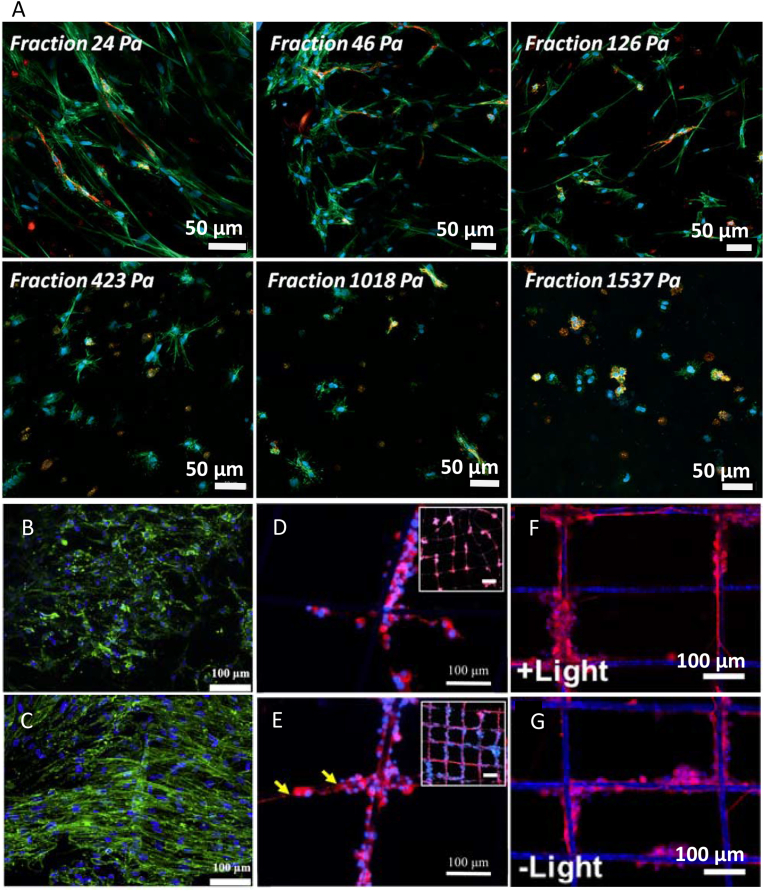
Confocal microscopy of the cells before and after the application of mechanical or electrical cues. (A) hAD-MSCs and HUVECs (green - actin filaments, red - CD31, blue - nuclei) encapsulated in GelMA gradient fractions (with different stiffness indicated on the images) after one week of cell culture. hSCs-laden FGelMA hydrogel (green - actin filaments, blue - nuclei) cultured under (B) static and (C) dynamic conditions. PC12 cells (red - tubulin, blue - nuclei) cultured on PCL fibers with 80 μm thick gold coating (D) without and (E) with electrical stimulation treatment after five days. Yellow arrows indicate neurite length. PC12 cells (red - tubulin, blue - nuclei) cultured on PCL/GO/g-C_3_N_4_ scaffold (F) with and (G) without light stimulation after one week. (A) Reproduced under the terms of the Creative Commons Attribution 4.0 International License from Ref. [133], Copyright © 2020 Wiley-VCH. (B, C) Reproduced under the terms of the Creative Commons Attribution NonCommercial License 4.0 from Ref. [134], Copyright © 2020 Elsevier. (D, E) Reproduced with permission from Ref. [141], Copyright © 2020 Elsevier. (F, G) Reproduced with permission from Ref. [91], Copyright © 2020 Elsevier.

Lavrentieva et al. [133] fabricated scaffolds with stiffness gradient using photoactive hydrogel GelMA. Encapsulated human adipose tissue-derived mesenchymal stem cells (hAD-MSCs) and HUVECs were co-cultured for seven days. The gradient in stiffness in the printed material was obtained by mixing while printing two compounds, namely, GelMA with higher and GelMA with lower methacrylation degree. A microfluidic device was used for homogenous mixing of the compounds. Six gradient fractions were obtained in the final scaffolds, with a mechanical stiffness ranging from 23.7 Pa to 1537 Pa. After seven days of culture, actin staining conﬁrmed decreased cellular spreading inside the material with increasing stiffness and no spreading for highest stiffness. The differences in the behavior of hAD-MSCs and HUVECs in the gradient fractions were noticed (Fig. 5A). HUVECs did not spread in fraction 125.6 Pa and higher. In contrast, some spreading of hAD-MSCs was observed even for the 423 Pa fraction [133].

Das et al. [69] utilized the 3D bioprinting technique to fabricate an engineered heart tissue. As the bioink, the authors used porcine heart-derived ECM or collagen with homogenously distributed cardiomyocytes. Scaffolds were prepared and cultured in static and dynamic (kept on an orbital shaker) conditions for 14 days. Samples cultured in the dynamic conditions revealed a unidirectional, rod-shaped, and extended alignment of embedded cardiomyocytes with a defined sarcomeric pattern. Moreover, they have shown abundant expression of cardiac-specific proteins and genes encoding the proteins responsible for cell-cell and cell-matrix interaction, such as anti-cardiac troponin T and *anti*-α-sarcomeric actinin antibody. The formation of basement proteins (laminins and integrins) and matrix remodeling events were higher in dynamic than static conditions. No apparent aligned sarcomeres and rounded morphologic cardiomyocytes were observed later. These results suggested that fluid shear stress can provide dynamic stimuli which enhance cardiomyocytes’ maturation due to the cell-to-cell contact and cell-matrix interaction [69].

In another study, the auxetic scaffolds with primary human Schwann cell-laden Fish gelatin meth-acrylamide (FGelMA) were printed for application in neural tissue engineering. To obtain an auxetic scaffold with the grid design, they used pluronic F127 as sacrificial polymer and GelMA to provide a frame for clamping. The effect of mechanotransduction on neural differentiation was investigated by applying to the sample tensile forces with the help of a tensile bioreactor (20% tensile strain, 0.48 Hz frequency). The scaffolds that underwent stimulation revealed enhanced cellular proliferation and differentiation; cells showed spindled-like extended neurite growth compared to static cultures (Fig. 5B and C). Application of the tensile forces together with other cues, such as growth factors, further enhanced neural differentiation [134].

Shear stress applied while printing was also shown to influence cell orientation in printed construct and stimulate regeneration of muscle tissue. Distler et al. [135] printed oxidized alginate/gelatin hydrogel with encapsulated C2C12 muscle cells using different printing nozzles (d = 250 μm – 330 μm) and pressures (30 kPa – 60 kPa). The high shear forces evoked cell orientation along the printed direction. The authors investigated the differentiation of C2C12 cells, cultured with horse serum enhancement, into myotubes and recognized the myofilament and sarcomere formation. The results indicated that the aligned growth of C2C12 cells is a significant contributor in achieving ordered and synchronized contractile muscle tissues [135]. Similarly, another group evaluated the effect of shear stress on printed grid-shaped strands with C2C12 encapsulated cells in GelMA and methacrylate collagen (ColMA) hydrogels. The cells were cultured for five days in bioinks before printing to induce cell elongation as elongated cells are more sensitive to the shear stress than the round-shaped cells. The cells in the printed construct revealed an oriented structure, and after 28 days of culture, a significant increase in myotube formation in the printing direction was observed. Furthermore, myogenic gene expression such as MyoD1, myogenin, myosin heavy chain was higher than those in non-precultured myoblasts-laden GelMA bioink [72].

##### 3.1.4.4 Summary

The changes in bioink stiffness influence cell morphology and differentiation. MSCs showed more spreading and migration on scaffolds with lower stiffness, an increase in scaffold stiffness decreased cell mobility in the scaffold. It was also demonstrated that MSCs cells differentiate into osteoblasts on more stiff scaffolds, while improved spreading of HUVECs was observed for softer scaffolds. The elastic properties of the scaffolds can also influence the behavior of cardiomyocytes which had higher contractions and maturation on more elastic scaffolds. The applied tension on scaffolds enhanced Schwann cells' proliferation and differentiation into the neural lineage. Moreover, the tension stimulation caused the alignment of cells in the direction of applied force. The shear stress during printing bioink with encapsulated C2C12 cells led to the oriented structure of cells, which supports muscle regeneration. These findings confirm the response of cells to the mechanical properties of the environment. Extrusion bioprinting, due to the possibility of bioink stiffness modification and shear stress applied to the cells during printing, is the method that uses mechanical cues to a great extent. A summary of the studies employing mechanical cues is presented in Table 4.

**Table 4:** Mechanical cues and their influence on cell behavior.

Printing method	Tissue	Cell cue	Main material(s)	Cell type(s)	Cell response	Ref.
**MEW**	Cardiac	Elastic properties of the scaffold	PCL	iPSC-CM	Enhanced level of cardiac markers and higher beating rate for softer scaffolds.	[43]
	Cartilage	Dynamic load	PCL/GelMA	Chondrocytes	Enhanced cartilage formation after dynamic loading.	[130]
**FDM**	Tendon	Constant tension	PU/PCL	hBMMSCs	Elongation of cells along the longitudinal axis of the applied tension.	[83]
**Extrusion printing**	Bone	Scaffold stiffness	Alginate/gelatin	hMSCs	Enhanced ALP activity and osteogenic differentiation on softer scaffolds.	[67]
	Neural	Dynamic tensile load	FGelMA	Schwann cells	Enhanced cell proliferation and neural differentiation after mechanical stimulation.	[134]
	Cardiac	Fluid shear stress	Porcine heart-derived ECM	Cardiomyocytes	The upregulated expression of cardiac-specific proteins and unidirectional, extended cell alignment after mechanical stimulation.	[69]
	Muscle	Shear stress	Oxidized alginate/gelatin	C2C12	Cell orientation along the printed direction for higher shear stresses in the nozzle. Increased myotubes differentiation.	[135]
		Shear stress	GelMA	C2C12	Increased myotube formation in the printing direction with increased myogenic gene expression for pre-cultured bioinks.	[72]
	Not specific	Scaffold stiffness	Alginate/gelatin	MSCs	Formation of the 3D interconnected cellular network and a more spread cell morphology in softer scaffolds.	[131]
		Scaffold stiffness	Alginate	L929	Improved cell migration and proliferation throughout the scaffold in softer scaffolds.	[132]
		Scaffold stiffness	GelMA	hAD-MSCs, HUVECs	Decreased cellular spreading and proliferation with increased scaffold stiffness.	[133]

#### 3.1.5 Electrical cues

Every cell has a membrane potential specific to the cell and tissue type. The addition of electrical cues can facilitate cell-cell interactions. The electrical cues were shown to influence cell migration and growth, and enhance cell adhesion [136], can be helpful during differentiation of stem cells into osteoblast lineage [137], and are particularly needed in nerve regeneration [138]. The electrical cues can be introduced via introducing the conductive particles into the scaffold matrix or grafting them on the surface of the scaffold [139].

##### 3.1.5.1 Near Field Electrospinning

Vijayavenkataraman et al. [63] performed *in vitro* neural differentiation studies using PC12 cells seeded on PCL/rGO (reduced graphene oxide) conductive scaffolds fabricated using the NFES approach. PCL/rGO material was obtained by adding rGO powder with PCL pellets into a 70% w/v acetic acid. Scaffolds printed with the composite material had lower mechanical properties than the pure PCL scaffolds, which is beneficial for neural differentiation. *In vitro* results revealed that the PCL/rGO scaffolds had shown significantly higher cell attachment and proliferation than the pure PCL scaffolds. This effect was assigned to the increased surface area of the rGO nanostructure. Furthermore, the RT-PCR studies revealed the significantly higher expression of the three crucial genes associated with neural differentiation, namely, β3-tubulin, NF–H, and GAP43 in the scaffolds printed with PCL/rGO. Also, in these samples, immunocytochemistry results showed higher expression of NF200 and β3-tubulin, and higher neurite outgrowth when compared to pure PCL scaffolds. The enhanced protein expression could result from cell-cell interactions. As cells communicate with each other by means of electrical signals, the addition of conductive particles facilitates the contact between them [63].

##### 3.1.5.2 Melt Electrowriting

Zhang et al. [140] created PCL scaffolds that combine topographic cues with electroactive cues to guide myoblast alignment and enhance differentiation into myotubes. To mimic the structure of skeletal muscle's ECM, MEW was used to print microscale parallel grooves on top of an aligned nanoscale fibrous electrospun mesh. A gold coating was applied to the electrospun mesh to introduce electroactive cues. Addition of the coating significantly enhanced the alignment of the myoblasts and the myotube formation due to improved cellular electric signal transferring. Different spacing (100 μm, 200 μm, and 300 μm) was proposed for myoblast formation, and an interfiber distance of 200 μm revealed the highest alignment and elongation of the cells [140]. In another study, Wang et al. [141] have produced MEW scaffolds with different thicknesses (0 nm – 80 nm) of gold nanolayer to evaluate neural differentiation of PC12 under electrical stimulation. The results have shown that an increase in gold coating thickness increases the differentiation of cells due to the easier transmission of electrical signals between cells. Furthermore, the neurite length on coated scaffolds was significantly higher than the uncoated PCL scaffold. The neural length increased with increased conductivity of scaffolds (i.e. coating thickness). Moreover, the length of neural cells on scaffolds with the thickest gold layer was 19.6-fold higher when electrical stimulation was applied than on the same scaffolds without electrical stimulation (Fig. 5D and E) [141].

In another study, MEW scaffolds were functionalized with graphene oxide (GO) nanosheets and graphitic carbon nitride nanoparticles (g-C₃N_4_) via electrostatic interactions and used for neural regeneration. To evaluate the effect of functionalization on PC12 cells growth, visible-light stimulation has been applied. Under the light stimulation, g-C₃N_4_ generates an electron–hole pairs, and electrons are transferred to the GO. The GO in turn transfers the electrons to the cells, which can be consider as indirect electrical stimulation. The length of neural cells was greater on scaffolds treated with light stimulation than those without stimulation (Fig. 5F and G). Most probably, this effect was caused by the activation of the reactive oxygen species (ROS) generated by g-C₃N_4_, as ROS are involved in the differentiation of cells into neurons [91].

##### 3.1.5.3 Fused Deposition Modeling

Electroactive scaffolds have been fabricated using extrusion 3D printing for application in bone tissue engineering by Wibowo et al. [142]. The authors prepared several ink formulations by mixing different amounts of conductive polyaniline (PANI) microparticles with the PCL. The conductivity of PCL/PANI scaffolds, measured by the four-point probe method, has increased significantly with the addition of 0.1 wt% PANI. Higher amounts of PANI showed only a small increase of conductivity compared to 0.1 wt%, and they revealed cytotoxic effects on hAD-MSCs. Cell proliferation was supported by pure PCL and PCL/0.1 wt% PANI, however, a higher proliferation rate was observed after 14 days of culture for the latter. The cells were also more spread and elongated on PCL/0.1 wt% PANI scaffolds. To conclude, the addition of PANI is beneficial for bone replacement as electric conductivity improves osseointegration [142].

Adams et al. [143] have fabricated a PCL scaffold for primary human cardiomyocytes (pHCM) cell culture. The scaffolds had a matrix shape with a porosity of 50%, with a strand size of 200 μm and 200 μm pores. The pHCM were seeded post-printing, and scaffolds were stimulated with electrical impulses (5 V, 2 ms pulses, 1 Hz) with the custom-built electric simulator device. Cells on stimulated scaffolds showed higher attachment in contrast to the unstimulated ones. 70% of seeded cells on scaffolds with electrical stimulation compared to only 4% of the cells on the unstimulated scaffolds exhibited cytoplasmic extensions. This might be due to the higher cellular differentiation induced by electrical stimulation revealed on stimulated scaffolds [143].

##### 3.1.5.4 Summary

The addition of conductive materials such as PANI or GO to the main ink's compound can increase the scaffold's conductivity. Other proposed approaches include scaffold coating with gold or grafting of conductive particles. Conductive cues can improve signal transmission between cells, thus improving cell proliferation and differentiation. The studies revealed that higher scaffold conductivity favored osseointegration and neural regeneration. Moreover, the neural cells tend to elongate and grow along an applied electric field during electrical stimulation. As conductive particles can usually withstand higher temperatures, electrical cues can be used relatively easily with all EBP techniques, including MEW and FDM. A summary of the studies employing electrical cues is presented in Table 5.

**Table 5:** Electrical cues and their influence on cell behavior.

Printing method	Tissue	Cell cue	Main material(s)	Cell type(s)	Cell response	Ref.
**NFES**	Neural	rGO	PCL	PC12	Higher cell attachment and proliferation, enhanced neural differentiation for scaffolds with rGO.	[63]
**MEW**	Neural	Gold coating, electrical stimulation	PCL	PC12	Increased neural differentiation with increased coating thickness. Improved neural length after electrical stimulation.	[141]
		GO/g-C₃N_4_, light stimulation	PCL	PC12	Improved neural length after light stimulation.	[91]
**MEW/electrospinning**	Muscle	Gold coating	PCL	Myoblasts	Enhanced alignment of myoblasts and myotube formation on coated scaffolds.	[140]
**FDM**	Bone	PANI	PCL	hAD-MSCs	More elongated cells on scaffolds with lower amounts of PANI.	[142]
	Cardiac	Electrical stimulation	PCL	pHCM	Higher cell attachment and differentiation with electrical stimulation.	[143]

#### 3.1.6 Surface roughness

The proliferation of cells, cell differentiation, and tissue formation depend on the initial interaction between cells and the scaffolds [144]. This interaction is strongly related to the surface topography, which influences initial cell adhesion [145]. Cells tend to attach easier to the rough surface over the smooth one, as the rough surface can adsorb more proteins necessary for cell adhesion, secreted by the cells or contained in the cell culture medium [146]. There are various methods allowing an increase in the surface roughness of printed scaffolds, including surface etching [147], plasma treatment [148,149], or enzymatic digestion [150].

##### 3.1.6.1 Near Field Electrospinning

**Fig. 6:**
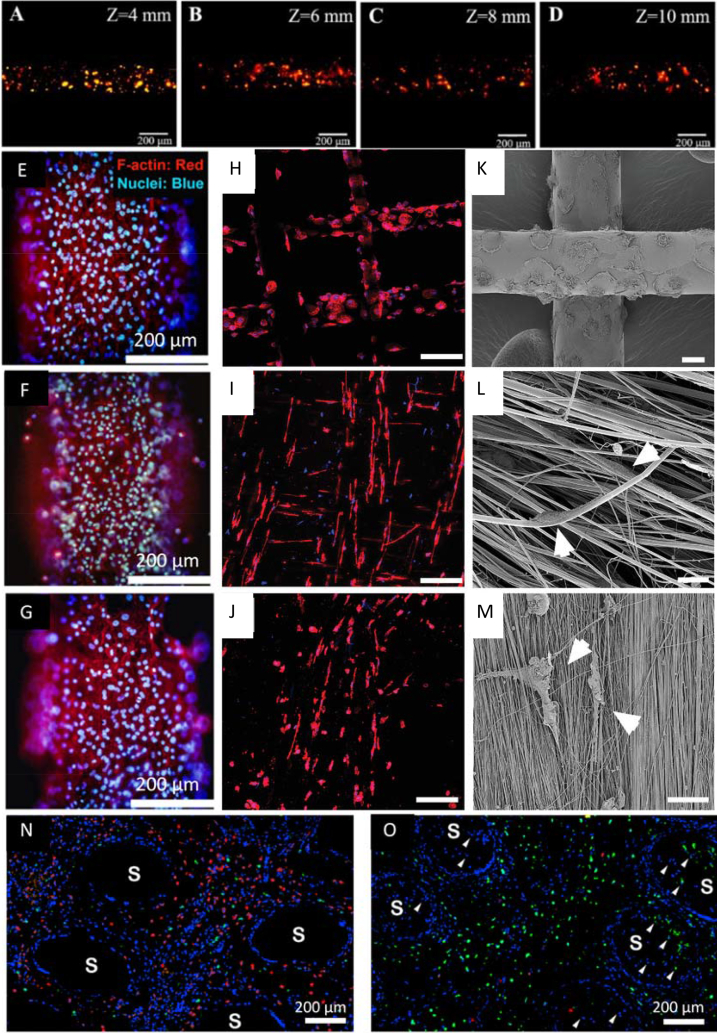
Effect of surface roughness and hierarchical structures on cells. Fluorescence images (the colors indicate live cells) of hMSCs after three days of culture on PCL NFES scaffolds printed with different distances between nozzle and collector (A) 4 mm, (B) 6 mm, (C) 8 mm, and (D) 10 mm. Chondrocytes cultured for seven days on PCL FDM scaffolds treated with a phosphate buffer solution at 25 °C for 30 min: (E) without enzyme, (F) with 5 mg/ml Novozyme®435, and (G) with 5 mg/ml Amano lipase PS (red - F-actin, blue - nuclei). Fluorescent (red- F-actin, blue-nuclei) and SEM images of macrophages after seven days culture on (H, K) PCL microfibers, (I, L) PCL nanofibrils, and (J, M) collagen scaffolds, respectively. The white arrows indicate elongated cells. Fluorescent images (red - M1 polarization marker CD11c, green - M2 polarization marker CD206, blue - nuclei) of MSCs after seven days of subcutaneous implantation in mice on (N) PLCL microfibers and (O) PLCL hierarchical porous scaffolds. The arrows indicate the infiltration of cells into the hierarchal scaffolds, S - scaffold. (A–D) Adapted under the terms of the Creative Commons Attribution License from Ref. [151], Copyright © 2020 MDPI. (E–G) Adapted with permission from Ref. [153], Copyright © 2019 Springer Nature. (H–M) Adapted with permission from Ref. [155], Copyright © 2021 Wiley-VCH. (N, O) Adapted with permission from Ref. [156], Copyright © 2021 Elsevier.

Jinag et al. [151] have printed the PCL square scaffolds (1 mm square pore size). The authors analyzed the influence of the nozzle to collector distance on the fiber roughness and consequent hMSCs attachment. The results have shown that with the nozzle–collector distance decreasing from 10 mm to 4 mm, the surface roughness was increasing due to the decreasing time for solvent evaporation before fiber deposition on the collector plate. More extensive changes in fiber morphology were observed because the fibers were more volatile and less resilient to the impact force during deposition. The *in vitro* studies revealed that more cells were attached and elongated on the scaffolds printed with higher roughness (distance between collector and the nozzle of 4 mm) (Fig. 6A–D) [151].

##### 3.1.6.2 Melt Electrowriting

PLLA scaffolds with 200 μm square pore size were printed using the MEW approach for bone tissue engineering. After printing, the scaffolds were functionalized using alkaline treatment (0.25 M or 0.5 M NaOH) for different periods of time (1 h–4 h) to improve the bioactivity of scaffolds. With the increasing time of alkaline treatment and concentration of NaOH, the surface roughness increased. This resulted in higher mouse osteoblastic cell (KUSA-A1) attachment and bone tissue formation, as revealed by deposition of mineral component, detected by FTIR and EDX. The authors explained the effect by the increase of hydrophilicity of the treated surface compared to the untreated PLLA surface (hydrophobic), which led to more suitable cell adhesion conditions. The hydrophilic properties of treated scaffolds were also the result of the ester functional groups introduced onto the surface during the treatment [86].

##### 3.1.6.3 Fused Deposition Modeling

Gupta et al. [152] have fabricated PCL scaffolds with 1 mm square pore sizes as a bone substitute, using the FDM approach. The authors have investigated the influence of NaOH treatment on the response of Saos2 osteosarcoma cells. An increase in fiber surface porosity and roughness was observed with the increasing time of NaOH treatment, the concentration of NaOH, and temperature. A reverse trend was observed for mechanical properties; namely, Young's modulus and compressive strength decreased with increasing time, temperature, and concentration of NaOH used for the treatment. The cells on the treated scaffolds developed more pseudopodia compared to the non-treated PCL scaffolds. The higher proliferation rates on treated samples were assigned to the increased hydrophilicity and surface roughness of that scaffolds. The authors concluded that the intensity of the NaOH treatment should be chosen carefully based on the desired mechanical and biological properties of the final material system. Since the treatment can be effectively performed irrespectively of the printed scaffold dimensions (as long as the NaOH solution can freely penetrate the structure), it can be applied to a broad range of patient-specific scaffolds with various designs [152].

A different approach to introduce surface roughness in the scaffolds for bone tissue engineering was suggested by Wang et al. [149]. The authors modified PLA scaffolds using cold atmospheric plasma to mimic the nanoscale ECM properties of bone tissue. The PLA scaffolds have been subjected to plasma treatment for 1 min, 3 min, and 5 min. The increase in roughness at the nano-level and hydrophilicity of the scaffolds was observed for increasing treatment time. Enhanced fibroblasts attachment was detected for all treated groups and was the most pronounced after 5-min treatment. Cell proliferation study conducted with osteoblast and MSCs has shown increased proliferation rates for the treated scaffolds, the highest for 1-min treatment at day five of culture. The improved cellular activity on plasma-treated scaffolds was associated with the nano roughness and change of the surface chemistry (formation of functional groups of oxygen and nitrogen) [149].

PCL scaffolds have been fabricated for cartilage tissue regeneration with a 0/90/45/135° laydown angle of adjacent layers and pore size of around 140 μm. To increase the hydrophilicity of the material, the scaffolds have been enzymatically hydrolyzed via immersion for different periods (10 min – 60 min) in the solution of bioenzyme: Novozyme®435 or Amano lipase PS. The roughness was observed in the treated scaffolds, increasing with the increase of the treatment temperature and the enzyme concentration. The attachment and proliferation of the seeded chondrocytes were higher on the enzyme-treated scaffolds (Fig. 6E–G). The cartilage-specific genes expression was significantly higher for treated scaffolds after 28 days. The optimal surface energy, roughness, and wettability for cell differentiation into chondrogenic lineage were observed for the 5 mg/ml concentration of Novozyme®435 enzyme used at 25 °C and 10 min – 60 min incubation time [153].

##### 3.1.6.4 Summary

The surface roughness of the fibrous scaffolds can be introduced by etching or degrading the polymers. The change in surface roughness is usually connected to the increased hydrophilicity of the scaffolds. The roughness can be controlled by the treatment parameters, e.g., temperature, time, concertation of the active agent. Alternating surface morphology is widely used for thermoplastics, typically lacking adhesive cell adhesion sites and forming smooth filaments. Those materials are also usually more stable in the treatment conditions than hydrogel-based systems. The studies have shown that the increase in surface roughness (and hydrophilicity) causes an increase in cell attachment and proliferation. The results also indicate that the mechanical properties deteriorate with the prolonged treatment time. Therefore, while introducing surface roughness, the possibility of change in mechanical properties should be considered and validated. A summary of the studies that utilize roughness as a cue is presented in Table 6.

**Table 6:** Surface roughness and its influence on cell behavior.

Printing method	Tissue	Cell cue	Main material(s)	Cell type(s)	Cell response	Ref.
**NFES**	Not specific	Surface roughness	PCL	hMSCs	Increased cell attachment and spreading on the scaffolds with higher surface roughness (printed with 4 mm needle – collector distance).	[151]
**MEW**	Bone	Surface roughness, hydrophilicity	PLLA	KUSA-A1	Increased amount of bone formation on scaffolds with higher surface roughness (treated with NaOH).	[86]
**FDM**	Bone	Surface roughness, hydrophilicity	PCL	Saos2 osteosarcoma cells	Enhanced proliferation rates on scaffolds with higher surface roughness (treated with NaOH).	[152]
		Surface roughness, hydrophilicity	PLA	Fibroblasts, osteoblasts, and MSCs	Increased cell attachment and proliferation on scaffolds with higher surface roughness (treated with cold atmospheric plasma).	[149]
	Cartilage	Surface roughness, hydrophilicity	PCL	Chondrocytes	Enhanced chondrogenic differentiation on scaffolds with higher surface roughness (after enzymatic treatment).	[153]

#### 3.1.7 Hierarchical structures

Native tissues and organs (e.g. skin, muscle, bone, or kidney) are characterized by complex and hierarchically organized structures at different length scales [154]. Some studies focused on the biomimetic approach and attempted to imitate the hierarchical assembly.

##### 3.1.7.1 Melt Electrowriting

Ryma et al. have used a MEW-based approach to obtain hierarchical fibrillar scaffolds, mimicking the assembly of collagen I. Two miscible blends were prepared: (1) poly(vinyl acetate) (PVAc) mixed with PCL at 70/30 wt% ratio, and (2) poly(2-*n*-propyl-2-oxazoline) (PnPrOx) mixed with poly(2-cyclopropyl-2-oxazoline) (PcycloPrOx)) at 30/70 wt% ratio, and extruded with a MEW printer to form a grid-shaped scaffold. Flow-directed phase separation of the mixed compounds was observed in both cases, and after the selective dissolution of one of the compounds, the nanofiber bundles were achieved. The human monocyte-derived macrophages seeded on the obtained hierarchical fibrillar scaffolds after seven days of culture infiltrated the meshes and showed elongated morphology in the direction of the nanofibrils, regardless of the blend used. In comparison, the macrophages seeded on traditionally MEW printed PCL and PnPrOx scaffolds (no hierarchical structure), and 2D films showed more rounded morphology. Additionally, the macrophages on PCL nanofibrillar hierarchical scaffolds showed M1 to M2 pro-healing polarization. A similar effect was observed in macrophages seeded on native rat collagen I nanofibrils (Fig. 6H-M). The authors concluded that the topological cues mimicking native ECM have the immunomodulatory capacity and can be used to introduce implants with prohealing capabilities [155].

##### 3.1.7.2 Fused Deposition Modeling

The hierarchical porous grid-shaped (1 mm interfiber distance) scaffolds were fabricated using the FDM approach with a cryogenic collector plate. In detail, poly (l-lactic acid-ε-caprolactone) (PLCL) mixed with nHAp (12 wt%) in 1,4-dioxane solution was printed on a cryogenic collector plate at −28 °C, and afterward immediately transferred to −80 °C for overnight storage. Finally, the scaffolds were lyophilized for 48 h to remove the residual solvent, which caused sponge bone-like hierarchical pores formation in the printed filaments (pore size 15.7 μm ± 6.3 μm). PLCL microfiber scaffolds without hierarchal pores were used as controls. The SEM results revealed that the hierarchical structures promoted MSCs cell adhesion at day one of the cell culture. Moreover, after seeding the lipopolysaccharide-stimulated macrophages on the hierarchical scaffolds with already seeded MSCs, the M1 polarization marker genes were significantly downregulated, while the M2 polarization marker genes were upregulated; no significant differences in gene expression of polarization markers were observed for conventional uniform scaffolds (Fig. 6N, O). The MSCs cultured on the hierarchical porous scaffolds together with osteoblasts and HUVECs enhanced osteogenic and angiogenic effects. In summary, the paracrine signaling of MSCs led to upregulation of the immunomodulatory, osteogenic, and angiogenic effects. *In vivo* studies revealed vascularized bone formation upon porous scaffolds implantation. The study indicated that hierarchical scaffolds lead to improved cell-material interaction due to the higher roughness and increased cell attachment area, promoting paracrine signaling of MSCs and facilitating the regeneration of damaged tissue [156].

##### 3.1.7.3 Extrusion bioprinting

Fan et al. [157] have printed muscle-like bundles using gelatin and fibrinogen with encapsulated C2C12 cells. They have fabricated scaffolds consisting of hydrogel-based parallel filaments forming bundles with various widths (the design of 0.6 mm, 2 mm, or 5 mm), hanging between supportive PDMS anchors. The myotube uniaxial orientation and myogenic differentiation gene expression levels were most pronounced in the 0.6 mm bundle design after day seven of culturing. In addition, Young's modulus increased from 15.42 kPa on day one to 147.67 kPa on day seven in that group due to myotube formation. The directional growth of myotubes was associated with the biomimetic design of the bundles that had dimensions and structure close to the native muscle tissue [157].

##### 3.1.7.4 Summary

Hierarchical fibrillar and multidimensional interconnected porous scaffolds induced a significant immunomodulatory M1-to-M2 switching mechanism in macrophages that resemble typical phenotype shifts in the healing process. Muscle-like bundles supported the directional growth of myotubes. These studies provide evidence that the architectural features of the native ECM offer clear guidance for the cells and can be used to introduce immunomodulatory capacity. Hierarchical nature-inspired structures can be translated into biomaterials to develop a potent biomimetic scaffold with a prohealing capacity for implantation. A summary of the studies using hierarchical structures is presented in Table 7.

**Table 7:** Hierarchical structures and their influence on cell behavior.

Printing method	Tissue	Cell cue	Main material(s)	Cell type(s)	Cell response	Ref.
**MEW**	Not specific	Hierarchical nanofibrillar scaffold	PVAc/PCL, PnPrOx/PcycloPrOx	Macrophages	M1 to M2 polarization on nanofibrillar scaffolds.	[155]
**FDM**	Bone	Hierarchical porous scaffolds	PLCL/nHAp	MSCs/macrophages/Osteoblasts/HUVECs	M1 to M2 polarization on hierarchical scaffolds.	[156]
**Extrusion printing**	Muscle	Hierarchical muscle-like bundles with various widths (0.6 mm, 2 mm, 5 mm)	Gelatin, fibrinogen	C2C12	Enhanced myogenic differentiation on bundles with a width of 0.6 mm.	[157]

### 3.2 Biochemical cues for cells

Biochemical cues include extracellular matrix integrated biomolecules such as proteins (i.e., fibronectin, vitronectin, laminin, cytokines), growth factors, enzymes, small cell-permeable molecules, drugs, and genetic regulators (large molecules) [159,160]. These molecules bind to the cell membrane receptors, activate the cellular signaling pathway, and alter gene expression in cells [161]. Compared with physical cues, biochemical cues are typically delivered in the form of soluble or insoluble factors admix to the ink composition [162].

#### 3.2.1 Substrate main composition

Inks composed of different materials aim to provide good printability and, at the same time, improve tissue-engineered scaffolds' biological functionalities by controlling components within the scaffold and matching tissue physiological characteristics.

##### 3.2.1.1 Melt Electrowriting

Bioprinted alginate methylcellulose (algMC) hydrogel scaffold was reinforced with MEW PCL fibers to increase the mechanical properties of the scaffolds. The pure algMC scaffold and the algMC/PCL hybrid material showed similar cytocompatibility; however, PCL scaffold combined with algMC hydrogel produced significantly higher amounts of sulfated glycosaminoglycan. The authors concluded that this hybrid system enhances the chondrogenesis of human chondrocytes due to the proper material stiffness [163].

##### 3.2.1.2 Fused Deposition Modeling

PCL/alginate scaffolds, with different amounts of alginate, were prepared with FDM for application in bone tissue engineering. To obtain a homogeneous distribution of alginate in PCL melt, alginate was mixed with PCL powder and regrounded prior to the melting. The mouse preosteoblast cell seeding efficiency increased from 24.9 ± 4.1% to 54.1 ± 2.5% for pure PCL and PCL with 30% alginate, respectively. The results can be explained by increased hydrophilicity of the scaffolds containing alginate. The number of live cells was also increased with an increased amount of alginate in scaffolds. Moreover, the level of cell differentiation was higher on the scaffolds containing alginate, as confirmed by upregulated levels of ALP and deposited mineralized ECM [164]. Melcova et al. [165] have fabricated scaffolds for bone tissue engineering of poly(3-hydroxybutyrate) (PHB)/PLA blends with the addition of different plasticizers, namely Citroflex (CT) and Syncroflex (SN). MSCs seeded on the scaffolds showed higher cell proliferation and viability on the scaffolds with SN compared to those with CT. The same trend was also noticed with increased expression of osteogenic markers ALP and RUNX2. The authors have concluded that PHB/PLA/SN scaffolds show promising potential in bone tissue regeneration as they have shown superior mechanical and biological performance in comparison to the scaffolds with CT [165].

##### 3.2.1.3 Extrusion bioprinting

Alginate is one of the most commonly used materials in extrusion bioprinting. Nevertheless, it does not present any cell-binding peptides that favor cell attachment [166]. To overcome this challenge, alginate-based scaffolds have been mixed with other materials to improve cell adhesion and proliferation. Jiao et al. [167] have fabricated alginate scaffolds with the addition of gelatin to investigate the behavior of encapsulated fibroblasts. The results have shown that the proliferation rate of cells increases with increasing amounts of gelatin. This is due to the presence of cell-binding motifs in the gelatin, which improve cell attachment compared to pure alginate scaffolds [167]. Lee et al. [168] have enriched sodium alginate scaffolds with different amounts of hyaluronic acid (HA) to culture encapsulated fibroblasts (NIH-3T3) for application in soft tissue engineering. The viability and proliferation of cells were higher for scaffolds containing HA. HA is one of the ECM's main components that play the role of signaling molecule for cell proliferation and migration [168]. Alginate scaffolds with different ratios of poly-l-lysine (PLL) or poly(glutamic acid) (PGA) were also shown to improve the cellular response of MG63. There was a significant increase in cell adhesion with an increase of PLL addition (0–1% w/v), while for PGA addition, the adhesion was improved at a similar extent regardless of the concentration (tested range: 0–2% w/v). The improved adhesion and proliferation on alginate/PLL scaffolds were assigned to the electrostatic interaction between PLL and cell membrane. In turn, the addition of PGA caused higher calcium deposition compared to the pure alginate scaffolds, which suggests osteogenic differentiation of cells. The effect can be explained by the presence of carboxyl groups in PGA, favoring apatite nucleation. There was no difference in calcium deposition between scaffolds with and without PLL [169].

Pati et al. [170] have developed dECM bioinks, composed of adipose- (adECM), cartilage- (cdECM), and heart- (hdECM) ECM. hAD-MSCs, human inferior turbinate-tissue-derived mesenchymal stromal cells (hTMSCs), and rat myoblast cells (L6) have been encapsulated in adECM, cdECM, and hdECM, respectively, to investigate the cell differentiation into a specific lineage. The results have shown that the maturation and differentiation of encapsulated cells into tissue-specific lineage was enhanced for the tissue-specific ECM-based boinks compared to the control scaffold prepared of collagen type I. These results indicate that the produced scaffolds can guide cells due to the unique composition of decellularized ECM [170]. In another study, bioinks consisting of collagen type I and HA with different ratios (2:1, 3:1, 4:1) have been fabricated for liver tissue engineering. The human hepatic stellate cell line (L × 2) or primary fetal activated hepatic stellate cells (aHSCs) were embedded in bioink to assess the cell behavior. Both cell types revealed high viability in all ink compositions. The morphology of Lx2 cells was the same, regardless of the boink used. Interestingly, the aHSCs cells were more elongated on scaffolds with collagen to HA ratio 3:1 and 4:1, indicating the increased cell-matrix interaction with increasing collagen type I content. Despite the greater elongation of cells on 4:1 scaffolds, the scaffolds with a ratio of 3:1 have been chosen as the optimal bioink due to the high collagen content (major ECM component) and stable printability [65].

##### 3.2.1.4 Summary

The bioink properties can be carefully tuned by using multicomponent systems to ensure good mechanical properties and good biocompatibility at the same time. Due to the poor cell adhesion of alginate scaffolds, gelatin and hyaluronic acid have been added to the ink. The addition of gelatin improves cell adhesion due to the inclusion of adhesive domains, whereas PLL improves cell seeding due to the electrostatic interaction between PLL and cell membrane. In turn, PGA-alginate scaffolds favor osteogenic differentiation. The studies also suggest that ECM derived from specific tissue can support differentiation of the stem cells into the specific related lineage. Using multicomponent inks is most often employed in extrusion bioprinting due to the ease of obtaining hydrogel composite materials. However, there are also studies in which multimaterial inks are used for MEW or FDM [118,164]. A summary of the studies employing substrate compositional cues is presented in Table 8.

**Table 8:** Substrate main composition and its influence on cell behavior.

Printing method	Tissue	Cell cue	Main material(s)	Cell type(s)	Cell response	Ref.
**MEW**	Cartilage	Substrate main composition	PCL/algMC hydrogel	Chondrocytes	Increased chondrogenesis on scaffolds with algMC.	[163]
**FDM**	Bone	Substrate main composition	PCL/alginate	MC3T3-E1	Increased seeding efficiency and osteoblast differentiation with increasing amount of alginate.	[164]
			PHB/PLA/SN, PHB/PLA/CT	MSCs	Induced osteogenic gene expression on PHB/PLA/CT scaffolds.	[165]
**Extrusion printing**	Liver	Substrate main composition	Collagen/hyaluronic acid	L × 2, aHSCs	Bigger elongation of cells on scaffolds with the increased collagen content.	[65]
	Various	Substrate main composition	Tissue-based decellularized extracellular matrix (dECM)	hAD-MSCs, hTMSCs, rat myoblast	Improved cell differentiation into tissue-based cell type on tissue-specific ECM.	[170]
	Not specific	Substrate main composition	Alginate/gelatin	Fibroblasts	Improved cell proliferation with increasing gelatin content.	[167]
		Substrate main composition	Sodium alginate/hyaluronic acid	NIH-3T3	Improved cell proliferation in the scaffolds with the addition of HA.	[168]
		Substrate main composition	Alginate/PLL/PGA	MG63	Increased adhesion and proliferation for scaffolds with PLL. Enhanced osteogenic differentiation for scaffolds with PGA.	[169]

#### 3.2.2 Polymer coatings

Biodegradable synthetic polymers like PLA, PCL and poly(D, l-lactide-*co*-glycolide) (PLGA) are often used for tissue engineering scaffolds fabrication by 3D printing technologies. Although they provide excellent mechanical support, the pure scaffolds lack bioactivity to promote cell adhesion [171,172]. Several coatings such as poly-l-lysine, poly-d-lysine, peptide motif responsible for cell adhesion (RGDs), fibronectin, or collagen ones were employed to induce scaffolds' bioactivity, specific cellular response or enhance cell adhesion and migration [[173], [174], [175], [176]].

##### 3.2.2.1 Melt Electrowriting

**Fig. 7:**
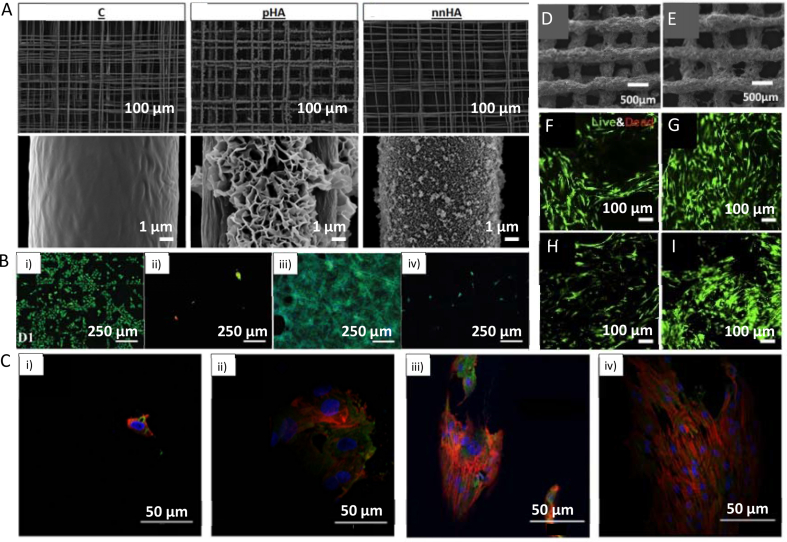
Examples of different coatings applied on the scaffolds. (A) SEM images of melt electrowritten scaffolds (from left to right): pure PCL (C) with plate-shaped micron-sized calcium phosphate crystals (pHA), and nano-needle hydroxyapatite (nnHA) coating. (B) Cell viability (green - live cells, red - dead cells) and cell morphology (blue - nuclei, green - cytoskeleton) on PCL MEW scaffolds after one day i, iii) with and ii, iv) without collagen coating, respectively. (C) Immunofluorescence images (green - vinculin, blue - nuclei, red - actin) of cells after day one on i) PLA, ii) Dopamine-coated PLA, iii) Collagen type I-coated PLA, iv) Dopamine-collagen-coated PLA scaffolds. SEM images of SF-based composite scaffolds (D) without and (E) with PRP treatment. Cell viability (green - live cells, red - dead cells) on SF-based scaffolds (F, G) after one week and (H, I) two weeks without and with PRP treatment, respectively. (A) Adapted with permission from Ref. [177], Copyright © 2020 Wiley-VCH. (B) Reproduced under the terms of the Creative Commons Attribution 4.0 International License from Ref. [179], Copyright © 2021 Wiley-VCH. (C) Reproduced with permission from Ref. [182], Copyright © 2018 Wiley-VCH. (D–I) Adapted under the terms of the Creative Commons Attribution NonCommercial License 4.0 from Ref. [64], Copyright © 2020 Elsevier.

PCL melt electrowritten scaffolds were fabricated to investigate the osteogenesis of MSCs. The scaffolds were coated with nano-needle hydroxyapatite (nnHAp), or plate HAp (pHAp) using the precipitation of simulated body fluid and Ca–P, respectively (Fig. 7A). Additionally, the scaffolds were functionalized with bone morphogenetic protein-2 (BMP-2) by adsorption. The authors concluded that PCL scaffolds with nnHAp coating significantly increase MSCs osteogenesis, as revealed by increased ALP activity and a controlled release of BMP-2 from the scaffold [177]. Another approach was implemented by Bertlein et al. [178], who coated PCL scaffolds (100 μm, 200 μm, and 350 μm square pore size) with ECM components such as fibronectin and gelatin to improve guidance of capillary structures formation. HUVECs formed thicker vascular structures on coated scaffolds due to the protein-enhanced cell adhesion and proliferation; the thickest one for 200 μm square pore size (the diffusion limit of nutrients is between 150 μm and 200 μm) [178]. In another study, PCL scaffolds were created to engineer human tympanic membrane. A collagen coating was applied to ensure airtightness on the PCL scaffold, composed of 4 layers with 45° laydown angle with an interfiber distance of 250 μm. Human keratinocytes (HaCaTs), seeded on top of the scaffolds, formed epithelial layers in scaffolds with and without the collagen coating. However, the coated scaffold had a significantly higher number of initially attached cells (Fig. 7B), and the scaffold's surface was entirely covered within seven days, which can be assigned to the collagen-containing scaffolds' higher surface area. The cells on collagen-coated scaffolds did not spread out uniformly over the collagen layer and did not align with PCL fibers. The crosslinking of collagen further increased the bending stiffness of the whole construct without having a negative effect on the cellular response [179]. In another study, the PCL scaffolds with 200 μm and 300 μm square pore size were coated with NCO-poly(ethylene oxide-*stat*-propylene oxide) (sP(EO-*stat*-PO)), and subsequently with collagen. The introduced sP(EO-stat-PO) coating increased surface hydrophilic properties and allowed the coupling of the collagen with the reactive –NCO groups. The hMSCs adhesion efficiency on the sP(EO-stat-PO)/collagen-coated scaffolds was comparable to the pure PCL. Although the authors concluded that there was no visible effect on cell adhesion between tested groups, the study showed promising potential in coupling other proteins, which can clearly lead to improved proliferation and differentiation into specific cell lineage [180]. ECM-based coatings, i.e, decellularized adipose tissue (DAP), fibronectin, and laminin, on PCL scaffolds (200 μm square pore size) were also used for the investigation of hMSCs adipogenic differentiation. The results have shown that all of the applied coatings supported cell adhesion and proliferation; yet, no significant differences in comparison to uncoated PCL scaffolds were observed. However, the adipogenic differentiation was improved and significantly higher on the scaffolds coated with DAP. Importantly, the effect of fibronectin and laminin alone on cell differentiation was lower. The authors assigned these observations to the fact that DAP closely mimics the properties of native ECM, consisting of more than 800 proteins, including fibronectin and laminin [181].

##### 3.2.2.2 Fused Deposition Modeling

PLA scaffolds were printed using FDM and coated with polydopamine (PDA), collagen type I (COL1), or both (PDA/COL1) to introduce adhesive cell sites and enhance the bioactivity of printed scaffolds. PLA printing provides high resolution and shape fidelity; however, the material has a hydrophobic nature which hinders cell adhesion. The ECM deposition and differentiation of BMSCs into osteogenic lineage were investigated on the samples with and without coating. The PDA/COL1 coating had increased cell adhesion, proliferation, and spreading (Fig. 7C). The use of PDA/COL1 coating enhanced the metabolic activity of BMSCs, expression of ALP, and deposition of ECM components after seven days of cell culture. The effect was observed due to the improved material hydrophilicity, introduction of functional groups (e.g. NH₂) of PDA, and cell adhesive domains present in collagen. The functional groups increased the adsorption of serum proteins from the cell culture medium, which supported cell adhesion. The higher levels of calcium and collagen deposition at day 14 of culture, observed for scaffolds coated with PDA/COL1, were related to coated collagen, an essential protein in the bone ECM [182]. Kovalcik et al. [183] have printed PLA and poly(3-hydroxybutyrate-*co*-3-hydroxyhexanoate) (PHBH) scaffolds. They have checked the cellular response of mouse embryonic fibroblast on the non-coated and gelatin-coated scaffolds. The PLA scaffolds coated with gelatin showed improved cell attachment and proliferation. In the case of PHBH, no significant effect of gelatin coating was observed, as pure PHBH already promotes cell growth (compared to PLA) due to its hydrophilicity [183]. PLA scaffolds were also coated with gelatin solution containing different amounts of mucic acid (PLA/GEL/MA) to investigate the osteogenesis of mouse mesenchymal stem cells (mMSCs). The upregulated levels of early and late-stage osteoblast differentiation markers were observed on PLA/GEL/MA scaffolds with a significant increase for scaffolds containing 10 μM of MA [184]. Cheng et al. [185] produced PLA scaffolds coated with PDA and grafted with BMP-2. The osteogenic differentiation of hMSCs on the produced scaffolds has been checked. The levels of early-stage markers of osteogenesis were significantly higher for scaffolds coated with PDA, with and without BMP-2. Nevertheless, the late-stage osteogenic markers were higher for scaffolds containing PDA and BMP-2. The differentiation of cells into osteogenic lineage was assigned to the release of grafted BMP-2 on the PDA-coated scaffolds, stimulating the proliferation and maturation of cells [185]. In another approach, PCL-printed scaffolds were coated with MSC-derived ECM. The coating has increased the hBMSCs adhesion and proliferation due to the presence of bioactive molecules in deposited decellularized-ECM. In the presence of the osteogenic induction medium, enhanced differentiation of cells into osteogenic lineage was observed on the coated scaffolds [186]. Also, the nanocellulose coating (CNF) on the PCL scaffolds was investigated by Rashad et al. [187]. The authors studied the potential differentiation of hBMSCs. The ALP activity, collagen type I production, and mineralization were upregulated in CNF-coated scaffolds compared to the untreated PCL scaffolds. The fibrillar structure of CNF stimulated the production of focal adhesion proteins, leading to cell spreading, elongation and consecutive differentiation into osteogenic lineage [187].

##### 3.2.2.3 Extrusion bioprinting

Wei et al. [64] have produced silk fibroin, gelatin, HA, and tricalcium phosphate (SF/GEL/HA/TCP) composite scaffolds using extrusion bioprinting. The scaffolds were coated with human platelet-rich plasma (PRP) to increase the proliferation and differentiation of seeded hAD-MSCs. Indeed, the cell proliferation was higher on scaffolds treated with PRP (Fig. 7D–I), most probably due to the presence of various growth factors in PRP. The results have also indicated that the PRP treatment supports late osteogenic stage markers (OCN, OPN) compared to the non-treated scaffolds. The upregulated level of those markers was possibly due to the higher density of cells favored by the PRP coating [64].

##### 3.2.2.4 Summary

The use of the coatings allows adjustment of the scaffold's cell-responsive properties without the need to change the ink composition and consequently without the need for printing parameters optimization. Conjugation of bioactive factors onto the fiber surface can be achieved chemically by amidation, esterification, or click reactions. However, it is not position-specific (small special resolution) and requires additional post-printing processing steps. In many cases, it excludes the use of bioink (inks with encapsulated living cells). The coatings of gelatin, collagen, or PDA improved cell attachment and proliferation. The scaffolds coated with MSC-derived ECM and human PRP contain multiple molecules which occur naturally in the tissue, enhancing the proliferation and differentiation of cells. Furthermore, nano-coatings of HAp or cellulose were used to improve cell behavior due to the increased hydrophilicity of scaffolds. Polymer coatings can be applied to scaffolds produced by any of the methods described. However, coatings are often used in scaffolds produced with FDM and MEW, as materials typically employed in these approaches lack adhesive proteins. A summary of the studies employing coating as a cue is presented in Table 9.

**Table 9:** Polymer coatings and their influence on cell behavior.

Printing method	Tissue	Cell cue	Main material(s)	Cell type(s)	Cell response	Ref.
**MEW**	Bone	nnHAp, or pHAp coating	PCL	MSCs	Enhanced osteogenic differentiation for coated scaffolds.	[177]
	Blood vein	Fibronectin, gelatin coating	PCL	HUVECs	Improved cell adhesion and proliferation on coated scaffolds.	[178]
	Tympanic membrane	Collagen coating	PCL	HaCaTs	Increased cell attachment and growth for coated scaffolds.	[179]
	Not specific	(sP(EO-stat-PO)) coating with coupled collagen	PCL	hMSCs	No visible differences on cell adhesion between coated and uncoated scaffolds.	[180]
		DAP, fibronectin, or laminin coating	PCL	hMSCs	Enhanced adipogenic differentiation on scaffolds coated with DAP.	[181]
**FDM**	Bone	PDA and collagen coating	PLA	BMSCs	Increased cell adhesion, proliferation, and osteogenic differentiation for coated scaffolds.	[182]
		Gelatin containing mucic acid coating	PLA	mMSCs	Enhanced osteogenic differentiation for coated scaffolds.	[184]
		PDA coating, BMP-2 grafting	PLA	hMSCs	Enhanced osteogenic differentiation for coated scaffolds with grafted BMP-2.	[185]
		MSC-derived ECM coating	PCL	hBMSCs	Upregulated bone-specific markers for coated scaffolds.	[186]
		Nanocellulose coating	PCL	hBMSCs	Enhanced ALP activity, collagen type I production, and mineralization for coated scaffolds.	[187]
	Not specific	Gelatin coating	PLA, PHBH	Mouse embryonic fibroblasts	Improved cell adhesion and proliferation on gelatin-coated PLA scaffolds.	[183]
**Extrusion printing**	Bone	Human platelet-rich plasma coating	SF/GEL/HA/TCP	hAD-MSCs	Upregulated levels of late osteogenic markers on coated scaffolds.	[64]

#### 3.2.3 Insoluble particles/molecules or immobilized cues

Insoluble particles and molecules can be incorporated into the ink composition before printing to elicit a specific cellular response in the fabricated material. The most commonly used cues within the EBP include HAp for bone tissue engineering [92], RGD for cell attachment, or GO for electrical conductivity and increase of surface roughness [63,188,189].

##### 3.2.3.1 Near Field Electrospinning

**Fig. 8:**
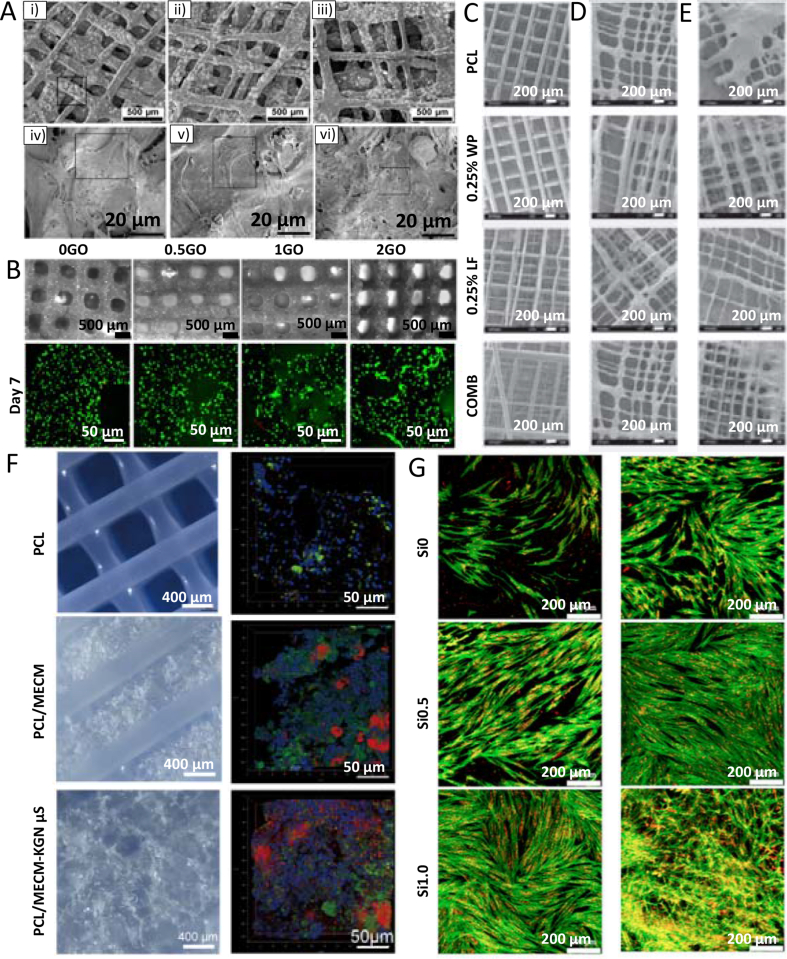
Cell behavior of scaffolds with insoluble and soluble cues. (A) SEM images of near field electrospun scaffolds and cell morphology on the scaffolds, containing different ratios of HAp: i, iv) PCL/0.3HAp, ii, v) PCL/0.4HAp, iii, vi) PCL/0.5HAp. (B) Light microscopy of cell-laden GO-containing scaffolds (top row) with cell viability (green - live cells, red - dead cells) after one week (bottom row). SEM images of PCL scaffolds with various amounts of WP, LP, and their combination (C) without cells, (D) after 14 days, and (E) after 21 days of cell culture. (F) Macroscopic images of PCL, PCL/meniscus extracellular matrix (MECM), and PCL/MECM-KGN scaffolds and immunofluorescent staining (blue - nuclei, red - CD105, green - CD73) of MSCs in implanted scaffolds four weeks after implantation (from left to right). (G) Immunofluorescence images (green - F-actin, red - collagen type I) of cells cultured on Si-GelMA hydrogels with different Si concentrations for (left column) three and (right column) seven days. (A) Adapted with permission from Ref. [92], Copyright © 2018 Elsevier. (B) Adapted under the terms of the Creative Commons Attribution NonCommercial License 4.0 from Ref. [204], Copyright © 2021 Elsevier. (C–E) Adapted with permission from Ref. [207], Copyright © 2019 IOP Publishing Ltd. (F) Adapted under the terms of the Creative Commons Attribution License (CC BY) from Ref. [209], Copyright © 2021 Frontiers Media S.A. (G) Adapted under the terms of the Creative Commons Attribution License from Ref. [212], Copyright © 2021 MDPI.

The use of the solvents in the NFES facilitates introducing additional particles into the polymer matrix. He et al. [92] fabricated scaffolds by combining PCL with HAp in HFIP at different ratios (HAp: PCL = 3:7, 4:6, 5:5) for bone substitute. Addition on the HAp remarkably increased the hydrophilicity of the constructs and increased MC3T3-E1 attachment, osteogenesis, and spreading. Cytoplasmic extensions were observed on all scaffolds with HAp addition (Fig. 8A). The gene analysis results indicated that the presence of HAp could induce the MC3T3-E1 cells to differentiate to osteoblast phenotype and enhance the ECM development and mineralization of pre-osteoblasts apatite surface. During *in vivo* studies with PCL/0.4HAp, a slight chronic inflammatory reaction was noticed. Formation of the new connective tissue and blood vessels was also observed [92]. Besides HAp, Kolan et al. [190] used PCL-B3 bioglass composite to mimic the native bone architecture. They obtained a microstructure similar to the cancellous bone, with ∼50% porosity and wide pore sizes distribution (20 μm – 250 μm). After seven days of culture, hAD-MSCs seeded post-printing showed a high proliferation rate. Interestingly, more dead cells were observed on PCL-B3 scaffolds than PCL-only scaffolds. The reason for relatively higher cell death in PCL-B3 glass scaffolds was the pH change induced by the B3 glass dissolution and, consequently, the release of ionic products, harmful for the cells [190].

##### 3.2.3.2 Fused Deposition Modeling

FDM enables the direct inclusion of the particles by mixing them with the ink. However, due to the use of higher temperatures, not every molecule can be added. HAp particles can withstand high temperatures (above 1000 °C [191]), so they can be easily incorporated into the printed material. In the study by Zhang et al. [192], PLLA scaffolds have been fabricated with 0 wt%, 30 wt%, and 50 wt% of nano-hydroxyapatite (nHAp) for application in bone regeneration. Rabbit mesenchymal stem cells (rMSCs) were seeded post-printing, and cell attachment and proliferation were analyzed. The data revealed that adding nHAp increased surface roughness, thus improving the scaffold's hydrophilicity. Consequently, cell attachment was higher on the rougher surface with more visible cell elongation than on the smooth PLLA surface. Four weeks after implantation, all scaffolds have shown good integration with the surrounding bone. However, a little bone formation was found in scaffolds with 30 wt% nHAp. Scaffolds with higher nHAp content (50%) revealed higher infill with new tissue, which indicates that higher nHAp content has better potential for the formation of new tissue [192]. It was also shown that the addition of 10 wt% nHAp to PLA scaffolds improved the proliferation of MG63, further favoring osteogenesis and osteoconductivity [193]. The effect of the HAp addition was also investigated for the scaffolds prepared from PLGA and PEEK. Both studies have reported that cell proliferation, ALP activity, and mineralization were higher for scaffolds containing HA compared to pure materials [194,195]. PCL/HAp scaffolds with varying ratios have also been used to treat bone defects. hMSCs were seeded on the printed scaffolds, and cellular differentiation and mineralization have been assessed. After day 28, cells were attached to all scaffolds. However, cells were more elongated and created confluent layers on scaffolds with increasing HAp amount due to increased hydrophilicity of the composite material. The upregulated level of bone markers (RUNX2, collagen type I, OPN, BSP) and the highest ALP level after 14 days of culture was observed on scaffolds with HAp concentration of 20% (w/w) and more. In summary, the results indicated that the differentiation of hMSCs toward osteogenic lineage was enhanced by the higher content of HAp [196]. Pierantozzi et al. [197] also suggested that adding strontium substituted HAp (SrHAp) to the polymer matrix enhances the mineralization and differentiation of hMSCs compared to pure PCL and PCL/HAp scaffolds [197]. In another study, PLA scaffolds were printed with the addition of varying bioglass (BG) content (up to 10 wt%). The *in vitro* study has shown no significant difference in MC3T3-E1 viability on scaffolds with different BG content. For the cells seeded on the PLA/BG scaffolds in osteogenic medium, higher expression of both osteoblast markers (ALP, RUNX2) and ECM markers (Col1) was observed compared to the pure PLA scaffolds. This was due to the synergistic effect of differentiation medium and bioglass which releases ions (e.g. silica and calcium [198]) responsible for osteostimulation [199]. Also, PVA scaffolds with the addition of the β-tricalcium phosphate (β-TCP) were fabricated for bone tissue engineering. The results indicated that scaffolds with higher content of β-TCP (20 wt%) exhibited higher mechanical properties and increased cell proliferation (ca. 1.67x) after four and seven days of cell culture [200].

Arastouei et al. [201] have fabricated PLA/akermanite scaffolds coated with gentamicin-loaded gelatin microspheres. Microspheres with gentamicin were added to provide antibacterial properties while akermanite guided cell behavior. The increasing amount of akermanite from 0 wt% to 30 wt% results in the higher cell viability, proliferation rate, and cell growth of MG63 cells. On the pure PLA scaffolds, cells had a spherical shape and fewer pseudopodia spreading areas than the akermanite scaffolds. That suggests that the surface of PLA/akermanite scaffolds favors the cell anchoring and spreading due to higher surface roughness. Calcium deposition increased with the increasing amount of akermanite (up to 20%; no statistical difference in deposited calcium for 20% and 30% of akermanite). The highest ALP activity was observed in the PLA/30% akermanite scaffolds. The authors concluded that the presence of Si and Ca ions in akermanite can stimulate the increase in mineralization of matrix and differentiation of cells [201]. Furthermore, Vyas et al. [202] have studied the PCL scaffolds with silk microparticles (SMP) for bone regeneration. SMP were added with varying concentrations (0–30 wt%) to enhance the mechanical properties and guide cell behavior, i.e. cell viability, morphology, and activity of hADSCs seeded on the scaffolds. The addition of SMP (up to 20 wt%) favored cell attachment and viability. Interestingly, the scaffolds with the highest concentration of SMP (30 wt%) showed a significant reduction of metabolic activity after seven days of culture. This effect can be associated with material cytotoxicity, which reduces cell number. The elongated cells on the scaffolds were observed only up to 10 wt% SMP, while more rounded cells were observed for 20 and 30 wt% SMP. The different cell morphologies can result from the increased scaffold stiffness and hydrophobicity caused by increased SMP concentration. The presence of calcium deposition was related to osteogenic differentiation of cells triggered by the presence of silk, causing nucleation and mineralization of calcium [202]. Seyedsalehi et al. [189] fabricated PCL scaffolds with rGO at concentrations up to 3 wt%. The growth and proliferation of hAD-MSCs were enhanced for scaffolds with rGO addition. Cells on scaffolds with 0.5 wt% and 1 wt% of rGO have shown better attachment to the fibers compared to the pure PCL scaffolds and were able to bridge the printed strands. The results indicate that the addition of rGO increases surface roughness and protein absorption, which leads to the improved cellular response on these scaffolds [189].

##### 3.2.3.3 Extrusion bioprinting

Extrusion bioprinting allows introducing the particles by mixing them with the main ink before printing. Due to no need for additional processing, many authors have used this method to produce bio-instructive scaffolds. However, the addition of the particles can change the rheological properties of the ink, requiring optimization of the ink composition and parameters of printing. In one of the studies, GelMA scaffolds were fabricated with different concentrations of Sr nanoparticles for application in bone tissue engineering. hMSCs were embedded in bioink before printing. Sr concentrations higher than 10 mg/ml resulted in reduced cell viability. The concentration of 1.5 mg/ml of nanoparticles revealed upregulated expression levels of ALP, collagen type I, and OCN expression after seven days of culture. That effect was associated with Sr ions which can compensate for calcium deficit in the cell culture medium [203]. The effect of GO addition to alginate/gelatin ink on the differentiation of hMSCs into osteoblasts was studied by Zhang et al. [204]. GO/alginate/gelatin scaffolds laden with hMSCs have been printed, and it was observed that the cells were viable and proliferated until day 42 of culture (Fig. 8B). The GO concentration was 0.5 mg/ml, 1 mg/ml and 2 mg/ml. The highest osteogenic differentiation was observed for the scaffolds with 1 mg/ml GO based on osteogenic-related gene expression. Incorporation of GO seemed to increase mineral content after 42 days of cell culture in a bioreactor, indicating the suitability of GO addition for bone defect treatment due to specific bioactive groups (e.g. OH– and COO–) and its absorption of serum proteins [204].

Incorporating cell adhesive domains was also used in printing approaches to improve cellular performance. For peripheral nerve tissue regeneration, Sarker et al. [188] has produced peptide-conjugated sodium alginate (PCSA) scaffolds. The authors have fabricated a 2%(w/v) alginate precursor with conjugates of RGD (RCSA), tyrosine-isoleucine-glycine-serine-arginine (YIGDR) (YCSA), and their mixture (composite PCSA). The processes of Schwan cells seeded on the scaffolds were significantly longer in the systems containing RGD, the longest for a composite of PCSA. In contrast, the cells on the non-modified alginate scaffolds had circular morphology. The brain-derived neurotrophic factor (BDNF) levels essential for neuronal survival and growth were the highest in scaffolds containing YIGDR. Nevertheless, the BDNF levels were lower compared to the control samples. Additionally, composite PCSA scaffolds have shown better outgrowth of neuronal cells in the direction of printed strands. Improved performance of cells on composite PCSA scaffolds was connected to the presence of RGD and YIGDR, which bind to integrin and laminin receptors of neuron cells, respectively, enhancing cell adhesion, growth, and proliferation [188].

Different amounts of RGDs were also covalently grafted to the alginate scaffolds to improve cell attachment and metabolic activity of human mammary fibroblasts (hMF) seeded on the scaffolds or epithelial cells encapsulated in the scaffolds. The cell attachment of hMF on scaffolds with RGDs was higher than on the non-modified ones. Scaffolds with RGDs also promoted the homogeneous production of ECM components (e.g. fibronectin network, collagen type I, and IV), on the scaffolds. This effect was due to the way of cell seeding. The hMF were cultured on top of hydrogel, which allowed cell spreading and ECM deposition without any physical barriers compared to the scaffolds with epithelial cells encapsulated in the hydrogel. The optimal concentration of RGDs was established at 400 μM, for cells seeded on top of the scaffolds, as it provides a good compromise between initial cell attachment and metabolic activity [205]. Hiller et al. [206] have fabricated scaffolds consisting of alginate-gelatin bioink with different amounts of human ECM (0 mg/ml, 0.25 mg/ml, 0.5 mg/ml, 1 mg/ml, and 2 mg/ml) for liver tissue engineering. Human HepaRG liver cells were embedded in the bioink prior to the printing to assess the influence of various ECM concentrations on cell viability and metabolic activity. After seven days of culture, cell viability was the highest for scaffolds containing 0.5 mg/ml and 1 mg/ml of ECM. These scaffolds have also improved hepatic metabolic activity due to the presence of hECM proteins [206].

##### 3.2.3.4 Summary

HAp was shown to be a bioactive material that improves the differentiation of stem cells into osteoblast lineage, enhances collagen and calcium deposition, and supports bone ingrowth after transplantation. Similar properties as HAp has bioglass and β-TCP, which also induce osteogenic differentiation. Furthermore, the other insoluble molecules such as silk microparticles, akermanite, or strontium substituted HAp has also favored bone regeneration. However, higher content of SMP (>30 wt%) led to cytotoxicity, which was not observed for the rest of the materials. GO has specific bioactive groups which enhance cell proliferation and mineral deposition. The addition of proteins improves cell seeding and thus cell proliferation and differentiation on the scaffolds due to the cell adhesive sites. The incorporation of ECM boosts cell proliferation due to its unique composition of proteins. Due to the ease of incorporation of particles into the ink, extrusion bioprinting is the most common method used to employ insoluble cues. Insoluble particles which can withstand higher temperatures are also used in FDM scaffolds. A summary of the studies employing insoluble cues is presented in Table 10.

**Table 10:** Insoluble cues and their influence on cell behavior.

Printing method	Tissue	Cell cue	Main material(s)	Cell type(s)	Cell response	Ref.
**NFES**	Bone	HAp	PCL	MC3T3-E1	Enhanced ECM deposition, mineralization, and osteoblast differentiation on scaffolds with HAp.	[92]
		B3 glass	PCL	hAD-MSCs	High rate of cell proliferation on scaffolds with B3 glass.	[190]
**FDM**	Bone	nHAp	PLLA	rMSCs	Improved osseointegration on scaffolds with nHAp.	[192]
		nHAp	PLA	MG63	Improved proliferation and osteogenic differentiation on scaffolds with nHAp.	[193]
		HAp	PLGA, PEEK	hAD-MSCs	Enhanced osteogenic differentiation on scaffolds with HAp.	[194,195]
		HAp	PCL	hMSCs	Enhanced osteogenic differentiation on scaffolds with HAp.	[196]
		SrHAp	PCL	hMSCs	Enhanced differentiation on scaffolds with SrHAp.	[197]
		Bioglass	PLA	MC3T3-E1	Upregulated levels of osteogenic and ECM markers on scaffolds with bioglass.	[199]
		β-TCP	PVA	L929 cells	Increased proliferation on scaffolds with β-TCP.	[200]
		Akermanite	PCL	MG63	Improved cell spreading and osteogenic differentiation on scaffolds with akermanite.	[201]
		SMP	PCL	hADSCs	Enhanced cell elongation and osteogenic differentiation for scaffolds with lower SMP concentration.	[202]
	Not specific	rGO	PCL	hAD-MSCs	Enhanced cell proliferation on scaffolds rGO.	[189]
**Extrusion printing**	Bone	Sr nanoparticles	GelMA	hMSCs	Upregulated levels of osteogenic markers for lower Sr concentrations.	[203]
		GO	Alginate/gelatin	hMSCs	Enhanced osteogenic differentiation for lower GO concentrations.	[204]
	Neural	RGDs, YIGDR	Alginate	Schwann cells	Improved adhesion and proliferation for scaffolds with both RGDs and YIGDR.	[188]
	Liver	ECM	Alginate/gelatin	Human HepaRG liver cells	Improved hepatic metabolic activity for lower ECM concentrations.	[206]
	Breast	RGDs	Alginate	hMF	Improved cell attachment and spreading, enhanced ECM production for scaffolds with RGDs.	[205]

#### 3.2.4 Soluble cues

Soluble cues are growth factors and derivatives, such as EGF, VEGF, FGF, and TGF β, or drugs that increase cells' paracrine signaling to accelerate tissue regeneration [162]. The soluble factors interact with cells through extracellular or intracellular mechanisms and stimulate cell response by cell signaling pathways [160].

##### 3.2.4.1 Melt Electrowriting

To increase the biological functionality of PCL scaffolds, Hewitt et al. [207] blended PCL with bioactive milk proteins (MPs). Two different MPs, whey protein (WP) and lactoferrin (LF), were mixed with concentrations of 0.05%, 0.01%, and 0.25%. Also, a combination of 0.25% of both WP and LF was used. HDFs and HaCaTs were used to examine the biological activity of released MPs. The scaffolds created of PCL blended with LF or an LF/WP combination showed significantly enhanced cell growth, spreading, and infiltration compared with untreated PCL scaffolds (Fig. 8C–E). The authors hypothesize that the scaffolds' enhanced tissue regeneration is due to the MPs' immunomodulatory effects, mainly the anti-oxidative effects of WP and the anti-inflammatory effects of LF. WP can directly enhance the production of GSH, an antioxidant molecule. LF can activate anti-inflammatory pathways, reducing inflammation. It is expected that LF and WP synergistically contribute to tissue regeneration. Therefore, blending PCL with LF and WP could potentially create constructs for deep tissue dermal regeneration [207].

##### 3.2.4.2 Fused Deposition Modeling

In another approach, Farto-Vaamonde et al. [208] designed and printed PLA scaffolds with prednisolone (Pred) and dexamethasone (Dex) drugs using the FDM method. They have checked two strategies: encapsulation of the drug in the filament prior to the printing and coating PLA scaffolds with a drug solution after printing. Double-loaded scaffolds were also studied where Dex was encapsulated inside the fibers, and Pred was coated on the scaffold. The scaffolds containing Dex inside the polymer matrix induced upregulated osteocalcin and ALP levels in hMSCs cell culture due to prolonged release of the osteogenic drug. Pred-loaded scaffolds after printing showed good anti-inflammatory properties. Moreover, double-loaded scaffolds revealed the possibility of gradient drugs release, as coated drugs release faster compared to the one encapsulated inside fibers [208]. Another study focused on scaffolds for meniscus tissue engineering. The PCL scaffolds were immersed in meniscus extracellular matrix (MECM) gel with or without kartogenin (KGN)-loaded PLGA microspheres. The results have shown that the presence of MECM has a positive effect on synovium-derived mesenchymal stem cells (SMSCs) adhesion and proliferation. Furthermore, the release of KGN from microparticles enhanced the chondrogenic differentiation of cells because of the prochondrogenic activity of KGN on cells. The *in vivo* studies on rabbits revealed that for all the scaffolds, except pure PCL constructs, the meniscus regeneration was more advanced four weeks post-implantation (Fig. 8F) [209]. In another study, biphasic scaffolds consisting of a PCL mesh (300 μm square pore size) and casted GelMA with encapsulated cells have been fabricated to regenerate cartilage tissue. A co-culture of BMSCs and costal chondrocytes (CChon) (3:1 ratio) within GelMA has been used. The TGF-β3 was added to hydrogel to facilitate cartilage regeneration further. *In vitro* studies revealed higher cartilage gene expression markers on scaffolds with TGF-β3, indicating that the growth factor plays a crucial role in stimulating cartilage formation. Furthermore, *in vivo* studies have also shown the elevated expression of cartilage-specific markers on scaffolds containing TGF-β3, higher neocartilage formation, collagen II deposition, and similar cell arrangement to natural cartilage tissue [210].

##### 3.2.4.3 Extrusion bioprinting

Multiple soluble particles and molecules have been proposed to guide cells in extrusion bioprinted scaffolds. HAp/chitosan/sodium hyaluronate composite scaffolds (CS) with loaded growth factors have been produced to treat bone defects. The scaffolds were immersed in solutions containing BMP-2 and vascular endothelial growth factors (VEGF). The effect of the release of growth factors on MC3T3-E1 cell proliferation and osteogenic activity has been investigated. Cell adhesion and proliferation were comparable on scaffolds with (CS + GF) and without growth factors (CS). However, the osteogenic differentiation and gene expression (OCN and Col1) were enhanced on CS + GF scaffolds. Moreover, *in vivo* studies 12 weeks after implantation revealed significantly higher new vascularized bone formation for these scaffolds. The effect can be explained by the ability to promote angiogenesis and bone regeneration by VEGF and BMP-2, respectively [211]. Si ions were incorporated into GelMA scaffolds for wound healing applications. The proliferation rate of HDFs seeded on scaffolds was higher for scaffolds rich in Si ions (highest proliferation rate detected for 1 mM of Si ions) (Fig. 8G). In addition, levels of pERK/ERK and pp38/p38 proteins, which are key factors in cell proliferation and differentiation, were upregulated compared to scaffolds without Si ions. The presence of Si ions in the hydrogels also caused an increase in the level of collagen type I and the expression of ECM remodeling-related biomarkers. The enhanced biocompatibility of GelMA scaffolds with Si ions was due to the constant release of Si ions and their availability to the cells on the hydrogel surface [212].

##### 3.2.4.4 Summary

The most commonly used mobile cues for cells are different growth factors. The release of a given growth factor causes faster cell proliferation and differentiation of stem cells to a given cell line, e.g. BMP-2 for osteogenesis and VEGF for vascularization. Apart from the growth factors, drugs are also very often used as mobile cues, and prolonged release of these molecules allows for more efficient tissue regeneration. Moreover, encapsulated drugs can have anti-inflammatory and antimicrobial properties, which are very important during implantation. Soluble cues are primarily used in extrusion bioprinting as hydrogel bioinks allow for migration of these particles and their release. A summary of the studies employing soluble cues is presented in Table 11.

**Table 11:** Soluble cues and their influence on cell behavior.

Printing method	Tissue	Cell cue	Main material(s)	Cell type(s)	Cell response	Ref.
**MEW**	Skin	Whey protein/lactoferrin	PCL	HDFs, HaCaTs	Enhanced cell growth, spreading, and infiltration on scaffolds with milk proteins.	[207]
**FDM**	Bone	Prednisolone and dexamethasone	PLA	hMSCs	Upregulated osteogenic markers levels and anti-inflammatory properties for scaffolds with Dex and Pred, respectively.	[208]
	Cartilage	Kartogenin (KGN)-loaded PLGA microspheres	PCL/MECM	SMSCs	Enhanced chondrogenic differentiation for scaffolds with drug-loaded microspheres.	[209]
		TGF-β3	PCL/GelMA	BMSCs, CCHon	Neocartilage tissue *in vivo* formation and higher collagen II deposition on scaffolds with TGF- β3.	[210]
**Extrusion printing**	Bone	BMP-2/VEGF	HAp/chitosan/sodium hyaluronate	MC3T3-E1	Enhanced osteogenic differentiation and *in vivo* vascularized bone formation on scaffolds with growth factors.	[211]
	Skin	Si ions	GelMA	HDFs	Improved cell proliferation and differentiation for the highest ion concentration.	[212]

#### 3.2.5 Co-culture

Co-culture is a method where two or more interacting cells are cultivated together. Co-culture systems provide the desired stimulus to facilitate cell viability and proliferation through the cell-to-cell signaling process (paracrine signaling) from one cell type to another [213]. This approach more closely represents human tissue as most tissues consist of multiple cell types. One of the good examples is human skin composed of stratified layers with different cell lineages. It was proved that the maturation of skin tissue is faster when co-culture is introduced to the scaffolds compared to monoculture [214]. Co-culture of MSCs with HUVECs on porous scaffolds has shown enhanced angiogenic paracrine activity (secretion and expression of angiogenic factors) and cell spreading [110]. The secretion of signaling molecules by one cell type affects adjacent cells, leading to improved tissue regeneration [214].

##### 3.2.5.1 Melt Electrowriting

PCL scaffolds with the addition of milk protein were used for co-culture of HaCaTs and HDFs cells in the skin model. The co-cultured cells have increased the wound gap closure rate compared to the monocultures due to the paracrine signaling. Fibroblasts produced growth factors and cytokines, which are responsible for keratinocyte stimulation during wound healing. Moreover, it was shown that the HaCaTs growth was increased in the presence of HDFs [207].

##### 3.2.5.2 Fused Deposition Modeling

Kuss et al. [215] have studied the effect of the co-culture of hAD-MSCs and HUVECs on vascularization and osteogenic differentiation. They have fabricated PCL/HAp composite scaffolds using FDM. The ADMSC-HUVECs were seeded directly on the scaffold surface, or the PCL/HAp scaffold was immersed in the hydrogel (HA-Gelatin) with encapsulated cells. The results have shown that the co-culture of ADMSC and HUVECs promotes cell migration and capillary networks formation due to upregulated levels of ECM degradation enzymes. Moreover, the presence of hAD-MSCs is essential for vascular network development because of the production of the pericyte maker responsible for vessel stabilization. The osteogenic differentiation of hAD-MSCs was not influenced by co-culture compared to the samples with monoculture [215].

##### 3.2.5.3 Extrusion bioprinting

In another study, Leucht et al. [216] have mixed methacryl-modified gelatin, non-modified gelatin, and acetylated methacryl-modified gelatin to produce a scaffold for vascular network formation using extrusion bioprinting. Co-cultured scaffolds consisted of vascular and osteogenic parts. The vascular part was encapsulated with hAD-MSCs and human dermal microvascular endothelial cells (HDMECs) whereas the osteogenic part was encapsulated with hAD-MSCs. The formation of vascular networks was enhanced in the co-cultured constructs with a higher total network length value than the control monoculture. The higher levels of osteogenic marker (OPN) in co-cultured scaffolds indicated the formation of a bone matrix. The improved vascularization was most probably caused by osteoblasts' secretion of VEGF. In turn, the HDMECs, in response to VEGF, released BMP-2, which induces osteogenesis [216].

Pourchet et al. [214] obtained mature skin tissue by two-step co-culture; firstly, they bioprinted the dermal layer with fibroblasts using bioink composed of gelatin, alginate, and fibrinogen. Secondly, they seeded keratinocytes on the top of the matured dermal layer. The dermal layer supported the formation of a stratified epithelial layer by providing neo-synthesized collagen and native physiological conditions [214]. Co-culture was also used for the human lung model. The alginate/gelatine/collagen scaffolds consisted of two parts. First, primary human lung fibroblasts (NHLFb) and monocytic THP-1 cells embedded in hydrogel were printed, followed by printing hydrogel with alveolar epithelial A549 cells. After 21 days, the A549 cells’ morphology changed to “egg-like” clusters (desired morphology in lung tissue), which was not observed for the monoculture of these cells. This effect was associated with the presence of fibroblast, which produces the ECM components in the lung, thus helping in the organization and polarization of the epithelial cells [217].

##### 3.2.5.4 Summary

Many tissues in the human body consist of more than one type of cell, each of which has different functions in the healthy tissue. Therefore, co-culture is gaining more and more interest in tissue engineering approaches. The data suggest that interactions between different cells are needed for faster and better tissue formation, as the cells secrete various proteins and markers that affect the behavior of other cells. Due to the possibility of incorporating the cells into the bioink, extrusion bioprinting is the most often used EBP approach to produce co-culture systems. A summary of the studies employing co-culture as a cue is presented in Table 12.

**Table 12:** Co-culture and its influence on cell behavior.

Printing method	Tissue	Cell cue	Main material(s)	Cell type(s)	Cell response	Ref.
**MEW**	Skin	Co-culture	Alginate/gelatin/fibrinogen	HaCaTs, HDFs	Formation of the stratified epithelium on a mature dermal layer during co-culture.	[207]
**FDM**	Bone	Co-culture	PCL/HAp/HA-Gelatin	hAD-MSCs, HUVECs	Formation of the vascular network during co-culture.	[215]
**Extrusion printing**	Bone	Co-culture	Gelatin	hAD-MSCs, HDMECs	Enhanced formation of the vascular network and osteogenic differentiation during co-culture.	[216]
	Skin	Co-culture	Gelatin, alginate, and fibrinogen	Fibroblasts, keratinocytes	Formation of the stratified epithelial layer during co-culture.	[214]
	Lung	Co-culture	Alginate/gelatin/collagen	NHLFb, THP-1, alveolar epithelial A549	The polarization of epithelial cells during co-culture.	[217]

### 3.3 Multiple cues

Tissue regeneration is a complex process, and introducing multiple cues simultaneously into a single system and tuning these cues to optimize for different tissues engineering remains challenging. Yet, this approach is also highly promising for obtaining fully functional scaffolds. Here, we discussed the studies which included multiple biochemical cues.

#### 3.3.1 Melt Electrowriting/inkjet printing

MEW was combined with inkjet printing to produce scaffolds with multiple (soluble and insoluble) cues. The scaffold consisted of three different zones, namely surface, middle, and deep layers, to closely mimic the gradient in cartilage tissue. Deep layers (100 layers, square pore size of 200 μm) were printed with the addition of HAp and then sprayed using inkjet printing with TGF-β1-loaded PLGA microspheres. 30 layers (200 μm square pore size) of PCL were printed to form the middle zone and sprayed with TGF-β1 and insulin-like growth factor-1 (IGF-1)-loaded microspheres. Finally, 20 layers (100 μm square pore size) of PCL were printed and sprayed with BMP-2 and TGF-β1-loaded microspheres for the surface zone. The results have shown that the combined release of multiple growth factors enhanced the chondrogenic differentiation of BMSCs in each zone. The combination of BMP-7 and TGF-β1 visibly elevated the expression of tissue-specific proteins indicating cartilage regeneration. This effect was further enhanced by the release of IGF-1 from microspheres. In addition, the incorporation of HAp into PCL matrix together with IGF-1, released from the microspheres, led to an increase in the RUNX expression promoting the regeneration of cartilage in the middle and surface zones. In turn, the combination of TGF-β1 and HAp led to the synergistic effect of higher collagen expression in the deep zone of the scaffold. The *in vivo* studies were performed on scaffolds without cells implanted in adult male New Zealand white rabbits. After six weeks, the gradient scaffolds filled in the injured site while the pure PCL scaffold showed poor cartilage regeneration. The results indicate that producing gradient scaffolds with the addition of growth factors present in a native tissue enhances the regeneration of this tissue [218]. In another study, the same research group used MEW to fabricate scaffolds with incorporated soluble (TGF-β1-loaded PLGA microspheres) and insoluble (HAp) cues for cartilage regeneration. The gelatin scaffold consisted of two layers: the top one with PLGA microspheres deposited with an inkjet dispenser to support articular cartilage repair, and the bottom one with the addition of HAp to the gelatin ink to promote bonding with the subchondral bone. Due to cytokine induction through soluble microspheres, BMSCs differentiated into chondrocytes and expressed cartilage-specific proteins in the top layer. The *in vivo* studies in rabbits revealed that after 24 weeks post-implantation, the damaged tissue was repaired with the gradient scaffold and provided good bonding with surrounding tissue, while for pure PCL scaffold, the damage was not fully repaired. The connection with the native bone was possible due to the synergistic effect of HAp and TGF-β1 addition, resulting in cell hypertrophy and increased collagen production [219].

#### 3.3.2 Fused Deposition Modeling

Tian et al. [220] have combined insoluble cue (HAp) and antimicrobial peptide (3-poly-l-lysine (EPL)) coating to produce scaffolds for bone tissue engineering. The addition of HAp caused the increase in roughness of the scaffold surface, which improved osteoblasts' (MC3T3-E1) attachment compared to the pure PCL scaffolds with a smooth surface. Moreover, the proliferation rate on the scaffolds with HAp and additional EPL coating increased in time and was significantly higher than on pure PCL scaffolds. Furthermore, enhanced osteogenesis and mineralization were observed on the scaffolds with multiple cues. The enhancement was connected to the combined effect of HAp inclusion and EPL coating, which simultaneously increased the surface roughness, hydrophilicity and introduced positive charges on the scaffold's surface [220]. Similar results were obtained for PHBH scaffolds with calcium sulfate hemihydrate (CaSH) addition coated with chitosan. In comparison to pure PHBH and uncoated PHBH/CaSH scaffolds, the coated systems promoted osteogenesis of rat bone marrow stromal cells (rBMSCs) with higher expressions of COL-1, BMP-2, or RUNX-2 and significantly higher ALP activity, indicating a synergistic effect of the cues. The addition of CaSH allowed for a more efficient chitosan coating, consequently strengthening the effect. *In vivo* studies showed good osteoinductivity of coated scaffolds four weeks post-implantation. In contrast, no, or minimal bone formation has been detected, respectively, for PHBH, and PHBH/CaSH scaffolds, further highlighting the synergy between cues [221].

#### 3.3.3 Summary

The approach of including multiple cues in one scaffold is still evolving in tissue engineering and regenerative medicine. Most scaffolds with multiple cues involve the addition of soluble or insoluble molecules to the main matrix. The different cues can be included in different layers, leading to the gradient hierarchical structures, closer imitating native tissues. The studies indicate the synergy between cues: the combined effect of multiple factors can lead to the improved cell adhesion, proliferation, or upregulated expressions of proteins necessary for the differentiation of cells into specific lineages, not visible or less pronounced in the scaffolds employing only one type of cell guidance strategy. The scaffolds with multiple cues are primarily used for cartilage and bone regeneration. It is essential to understand the cell response to single signals while producing scaffolds of higher complexity. A summary of the studies employing multiple cues is presented in Table 13.

**Table 13:** Multiple cues and their influence on cell behavior.

Printing method	Tissue	Cell cues	Main material(s)	Cell type(s)	Cell response	Ref.
**MEW/Inkjet printing**	Cartilage	Insoluble molecules (HAp), soluble factors (BMP-2, IGF-1, TGF-β1-loaded microspheres)	PCL	BMSCs	Enhanced chondrogenesis for cytokine-loaded scaffolds.	[218]
		Insoluble molecules (HAp), soluble factors (TGF-β1-loaded microspheres)	Gelatin/PLGA	BMSCs	Enhanced *in vivo* cartilage repair for cytokine-loaded scaffolds.	[219]
**FDM**	Bone	Insoluble molecules (HAp), coating (EPL)	PCL	MC3T3-E1	Enhanced cell proliferation and osteogenic differentiation on scaffolds with HAp and coating.	[220]
		Insoluble molecules (CaSH), coating (chitosan)	PHBH	rBMSCs	Enhanced osteogenic differentiation and *in vivo* bone formation for scaffolds with CaSH and coating.	[221]

### 3.4 Gradient scaffolds

Scaffolds with gradients in material composition, structure, mechanical characteristics, and biochemical or biological properties can closely recapitulate the structural and biological features of native hierarchical tissues [222]. Gradient scaffolds can provide porosity suitable for vascularization, efficient gas and waste diffusion, and nutrient supply with minimally reduced mechanical stability [223]. They can also guide the cells via providing varying, site-specific amounts of the particular cue or signaling molecule. It was, for example, shown that gradient in growth factors concentration supports construct vascularization [224], a gradient in stiffness supports cell differentiation [225], and changing spatial mineral distribution is beneficial for tendon-bone interface engineering [226]. Gradients can be obtained with the use of multiple extruders [227], alternation in printing parameters [228,229], or combining multiple EBP approaches [126].

#### 3.4.1 Melt Electrowriting

**Fig. 9:**
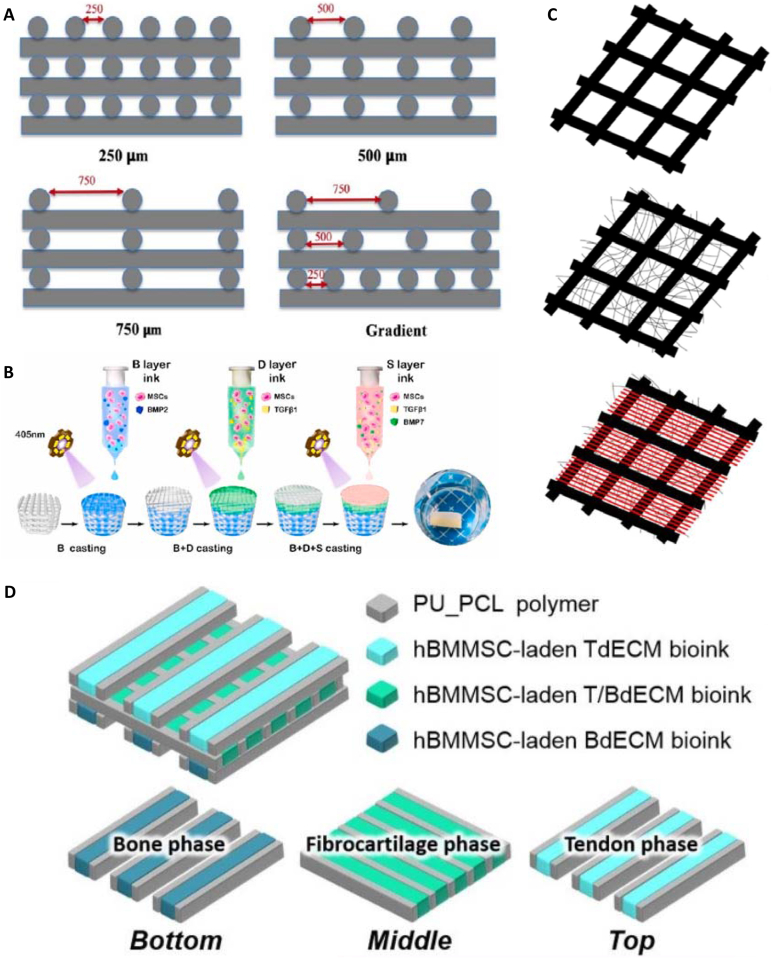
Schematics of gradient scaffolds. (A) Homogeneous pore size scaffolds (250 μm, 500 μm, 750 μm), and gradient PCL scaffolds. (B) Schematics of PCL-reinforced hydrogel scaffold. The hydrogel was mixed with MSCs with the addition of BMP-2, TGF-β1, or both BMP-2 and TGF-β1 to produce a gradient scaffold with three different layers. (C) Schematics of gradient scaffolds fabricated by combining different EBP methods (from top to bottom) FDM/SES, FDM/MEW, and FDM/SES/MEW. (D) The fabrication process of 3D cell-printed gradient scaffolds for the tendon-bone interface. (A) Adapted with permission from Ref. [228], Copyright © 2019 American Chemical Society. (B) Reproduced with permission from Ref. [85], Copyright © 2021 Elsevier. (C) Adapted with permission from Ref. [126], Copyright © 2021 IOP Publishing Ltd. (D) Adapted with permission from Ref. [83], Copyright © 2021 IOP Publishing Ltd.

MEW offers the possibility of printing precise shapes, pore and fiber sizes with high design flexibility. Therefore, MEW allows to produce scaffolds with well-controlled gradient structures. Abbasi et al. [228] have fabricated a three-layered PCL gradient scaffold consisting of the top, middle, and bottom layers with pore sizes of 750 μm, 500 μm, and 250 μm, respectively. Homogenous scaffolds were fabricated as controls (square pore size of 250 μm, 500 μm, and 750 μm) (Fig. 9A). The scaffolds were coated with calcium phosphate (CaP), and human osteoblasts were seeded. On day one, the homogenous scaffold with 250 μm pore size showed the highest initial attachment of osteoblasts. However, the gradient scaffold led to the highest amount of cell proliferation and infiltration at day 30 of cell culture. The results showed that gradient scaffolds have properties that can enhance cell growth and infiltration, caused by the increased surface area and stiffness [228]. In the follow up studies, the authors examined the *in vivo* osteoconductive capacity of these scaffolds. The scaffolds were implanted into mature female Wistar rats in the calvarial defects. Eight weeks after implantation, only gradient scaffolds (with the smallest pore sizes in the top layer) allowed for complete bone healing. Furthermore, the highest intensity of osteocalcin, a mineralization marker, was observed in gradient scaffolds and homogeneous scaffolds with 500 μm size pores. Additionally, a rise in the expression of endothelial markers was observed in gradient scaffolds, indicating that the repair process contained angiogenesis. In conclusion, the scaffolds with a properly chosen gradient pore size create excellent circumstances for bone regeneration due to a high permeability for oxygen and nutrients provided by larger pores and sufficient cell support and mechanical stability of the scaffold ensured by the inclusion of smaller pores [229]. In another study, Qiao et al. [85] fabricated a tri-layered stratified scaffold for osteochondral regeneration, inspired by the native gradient in collagen fiber architecture and ECM composition. The scaffold was composed of subchondral bone (B), deep cartilage (D), and superficial cartilage (S) zones with estimated porosities of 60.46%, 92.27%, and 86.89%, respectively (Fig. 9B). The scaffolds consisted of PCEC mesh produced with MEW and cast GelMA. Prior to the hydrogel casting, MSCs and layer-specific growth factors, namely, BMP-2 (B zone), TGF-β1 (D zone), and TGF-β1 with BMP-7 (S zone), were added to the hydrogel. Crosslinking of casted GelMA was performed using UV light. The results have shown that the MSCs could differentiate toward osteogenic lineage in zone B and chondrogenic lineage in zones S and D, resembling the cellular composition of the native osteochondral tissue [85].

#### 3.4.2 Fused Deposition Modeling/Melt Electrowriting/solution electrospinning

For the production of gradient scaffolds, two or more printing techniques can be combined. In the study by Wang et al. [126], MEW, SES, and FDM were used to produce multi-scale hierarchical scaffolds (FDM/SES/MEW) for tissue engineering (Fig. 9C). With FDM, the scaffolds with microfibers (diameters of several hundred micrometers) were produced. PCL/collagen solution was used to electrospun nanofibers on top of the FDM scaffold. Finally, MEW microfibers were printed on the electrospun mesh. As a control, scaffolds produced by combining FDM with SES (FDM/SES) and FDM with MEW (FDM/MEW) have been used. HUVECs and C2C12 cells were seeded in monocultures on different scaffolds to assess cell viability and proliferation. The proliferation rate after seven days was enhanced for both cell types on all the scaffolds compared to day one, with a higher proliferation of C2C12 cells compared to HUVECs. The improved attachment of cells was observed for scaffolds containing the SES layer due to the small pore sizes and high surface area, preventing cells from falling through the scaffold. Additionally, the presence of collagen in the SES layer further promoted the viability of cells and cell migration. Both types of cells were aligned and stretched along melt-electro written fibers for FDM/SES/MEW and FDM/MEW scaffolds, while random alignment was observed for FDM/SES scaffolds. The multiscale hierarchical scaffolds improved cell adhesion and proliferation, directing cell alignment through MEW scaffolds. Scaffolds produced by FDM provided the highest mechanical properties [126].

#### 3.4.3 Fused Deposition Modeling/extrusion bioprinting

FDM combined with extrusion bioprinting was used to print spatially-graded scaffolds for a tendon-bone interface. The scaffold consisted of three layers mimicking bone (bottom layer), fibrocartilage (middle layer), and tendon (top layer). PU/PCL scaffolds with pore sizes of ∼500 μm – 600 μm were printed using FDM to provide mechanical support for each layer. Extrusion bioprinting was used to print hBMMSCs-laden tendon-derived decellularized extracellular matrix (TdECM), bone decellularized extracellular matrix (BdECM), and their mixture (T/BdECM) bioinks between PU/PCL frame. To obtain a gradient scaffold, BdECM bioink was deposited and crosslinked between the bottom layers of the PU/PCL frame, T/BdECM bioink between middle layers, and TdECM bioink between top layers. (Fig. 9D). Cells have proliferated well in all the zones after 14 days of culture. Tenogenic markers were upregulated in TdECM bioink, indicating that the material promotes differentiation of hBMMSc into the tendon-specific lineage. In contrast, in the BdECM layer, osteogenic markers had significantly higher levels, indicating the induced osteogenic differentiation. The higher collagen type II and aggrecan content in the middle zone (T/BdECM) indicated the cell differentiation into a chondrogenic lineage. Different behavior of encapsulated cells within different zones was caused by the presence of tissue-specific matrix components together with signaling molecules and growth factors [83]. Y. Sun et al. [230] investigated the molecular mechanism underlying the regeneration of anisotropic cartilage by bioprinting rBMSCs encapsulated in the composite hydrogel (mixture of gelatin, fibrinogen, HA, and glycerol) between PCL fiber framework. Gradient scaffolds were composed of consecutively printed four layers, each 200 μm thick, with increasing pore sizes: 150 μm - the bottom layer, 350 μm and 550 μm – the middle layers, and 750 μm - the top layer. Homogeneous scaffolds with 150 μm and 750 μm pore sizes were used as controls. For gradient scaffold, chondrogenic differentiation and gradient cartilage matrix formation with a zone-specific expression of cartilage markers were observed but not detected for homogenous scaffolds. The results indicate that tissue regeneration can be further enhanced by producing gradient scaffolds [230].

#### 3.4.4 Summary

The scaffold architecture gradient allowed better cell infiltration and proliferation. Scaffolds with composition gradients led to the differentiation of stem cells into different lineages. The gradient in dECM content enhanced the differentiation of cells into osteogenic and chondrogenic lineage for interfacial tissue engineering. Scaffolds with zones of varying fiber thicknesses can be produced using different EBP methods or MEW alone (high control over the thickness of deposited fiber during a single print). Such scaffolds revealed improved cell seeding efficiency, proliferation, and cell alignment. Gradient scaffolds in composition could be produced by changing the bioink. A summary of the studies employing gradient scaffolds is presented in Table 14.

**Table 14:** Gradient scaffolds and their influence on cell behavior.

Printing method	Tissue	Cell cue	Main material(s)	Cell type(s)	Cell response	Ref.
**MEW**	Bone	Gradient in porosity (250 μm, 500 μm, and 750 μm pore sizes)	PCL	Osteoblasts	Enhanced cell proliferation and complete bone healing eight weeks after implantation for gradient scaffolds.	[228,229]
		Gradient in porosity, incorporation of MBP2, TGFβ1, BMP7	PCEC/GelMA	MSCs	Cell differentiation dependent on the incorporated growth factor.	[85]
**FDM/MEW/SE**	Not specific	Gradient in fiber thickness and alignment	PCL/collagen	HUVECs, C2C12	Improved cell adhesion, proliferation, and alignment on hierarchical scaffolds.	[126]
**FDM/Extrusion printing**	Tendon-to-bone	Gradient in growth factors	PU/PCL, TdECM/BdECM bioink	hBMSCs	Enhanced tenogenic, osteogenic, and chondrogenic differentiation within zones with tissue-specific matrix components.	[83]
	Cartilage	Gradient in pore sizes (150 μm–750 μm)	PCL, Gelatin/fibrinogen/HA/glycerol	rBMSCs	Induced heterogeneous chondrogenic differentiation and gradient cartilage formation for gradient scaffolds.	[230]

## 4 Applications of bio-instructive materials

3D EBP methods for printing bio-instructive materials have a wide range of applications, particularly in tissue engineering and regenerative medicine. The produced scaffolds are used to regenerate and build engineered constructs of various tissues, from hard to soft ones.

### 4.1 Bone

Bone tissue engineering is a leading field employing bio-instructive materials, especially based on FDM of PCL and PLA, which have high strength and slow degradation rates [119,164,182]. To enhance the osteogenic potential of printed scaffolds, biochemical cues, namely insoluble particles, such as HAp, are incorporated. Extrusion bioprinting and FDM are mainly chosen for printing with insoluble cues. Studies have shown that HAp strengthens the whole scaffold while ensuring improved osteoconductivity and good integration with neighboring bone [92,192,193]. Moreover, materials such as Sr particles, bioglass, or β-TCP are also incorporated into scaffold matrices to enhance bone formation [190,197,200]. MEW and FDM are used to produce bone substitutes with physical cues such as adjusted pore shape and size [107,108,[112], [113], [114],^119^]. For better cell adhesion and proliferation, polymer coatings are used on FDM scaffolds [[185], [186], [187]]. A couple of studies have suggested that the vascularization and bone formation processes can be enhanced with the co-culture of endothelial cells and stem cells [215,216].

### 4.2 Cartilage

For cartilage tissue regeneration, FDM and MEW were employed to print fibers reinforcing different hydrogel matrices [130,163]. The use of hydrogels allows incorporating the soluble cues such as growth factors, cartilage-based ECM, or proteins within scaffolds which improves the regeneration [209,218,219]. In addition, few studies have suggested that the rhombus pore shape can improve chondrogenic differentiation [121,122]. As cartilage protects bone, some researchers have produced gradient scaffolds mimicking both cartilage and bone zones for better integration with the surrounding tissue. HAp was used as an insoluble cue in the bone mimicking zone [218,219].

### 4.3 Cardiac tissue

For heart tissue engineering, mechanical cues favor cell contractions and maturation. Studies have shown that by choosing scaffold design with enhanced elastic properties or culturing cells in dynamic conditions, the levels of cardiac markers are upregulated [43,69]. Extrusion bioprinting is the leading method used for scaffolds in heart tissue regeneration. However, recently MEW also became attractive for that application due to the possibility of printing flexible scaffolds and new option to print hydrogels [118].

### 4.4 Neural tissue

The leading cue used for neural regeneration is electric stimulation. This cue is introduced in the form of a conductive coating or conductive materials (e.g. GO or gold), which increase the differentiation and the elongation of neural cells [63]. Electric stimulation amplifies these effects [91,141]. It was proved that cells tend to grow along the scaffold's longitudinal axis, which has a positive impact on neural differentiation [188]. The differentiation can be further improved by applying mechanical cues (e.g. tensile force) during *in vitro* studies [134].

### 4.5 Other tissues

Extrusion bioprinting is the most used method for the regeneration of soft tissues (e.g. skin, muscle, or liver), as hydrogel printing allows obtaining scaffolds with relevant stiffness. Alginate and gelatin are well-established biocompatible bioinks, with stiffness control provided e.g. by means of the amount of crosslinker added [67,[131], [132], [133]]. The regeneration of skin can be improved mainly by using soluble cues, which enhance cell proliferation and differentiation [207,212]. In turn, mechanical stimulation proved to be beneficial for tendon and muscle regeneration as it helps direct cells toward applied force [72,83,135]. The myotube formation can be further enhanced by electrical stimulation [140]. For lung regeneration, co-culture can be a critical cue due to the secretion of the essential signaling molecules by cells, as there are over 40 different types of cells in the lungs [217,231].

## 5 Future outlook

The fabrication of bio-instructive materials with EBP approaches can recapitulate the complexity of native tissues and advance tissue engineering strategies toward clinical translation. In this chapter, we discuss the future perspectives on how to boost that potential further.

### 5.1 Integrating multiple cues toward biomimicry

The extracellular microenvironment is a dynamic and changing environment that provides biophysical and biochemical cues which regulate cell behavior [232]. Integrating multiple cues into one biomaterial to provide a characteristic set of stimuli present in ECM is a promising approach for regenerating the native cell niche. Due to technological advancement in additive manufacturing, scaffolds with dynamic biophysical and biochemical cues might be brought up to a surprisingly superior level in the organ or tissue biomimicry and bring a revolution in primary research and regenerative medicine applications. For instance, Du et al. [233] developed a 3D scaffold that integrated several biophysical and biochemical cues (interconnected pore architecture, high porosity, high mechanical properties, rough surface, piezoelectricity property, and silica cross-linking network) for bone tissue regeneration. The biomimetic scaffold significantly enhanced cell attachment, proliferation, ECM mineralization, osteogenic gene expression *in vitro* and promoted *in vivo* bone regeneration [233]. Cheng et al. [234] also included multiple biophysical and chemical cues into the injectable silk-based bioink for osteochondral regeneration, such as the nanofibrous structures (silk nanofibers (SNF)), HA nanoparticles, and deferoxamine and BMP-2 molecules as angiogenic and osteogenic inducing factors, respectively. The produced materials were injectable and enhanced bone regeneration in *in vitro* and *in vivo* studies when compared to simple SNF/HA scaffolds without differentiation factors [234]. The inclusion of dynamic mechanical and electrical stimulations and different biochemical factors and topographies in one material system fabrication of new biomimetic biomaterials. We envision that the materials with increasing complexity and multidimensionality will gain a lot of interest in the coming years.

### 5.2 Hybrid living materials

The other field that has a great potential to benefit the development of bio-instructive materials are hybrid living materials. Those materials are composed of living organisms (e.g. cells, bacteria, microalgae, or yeast) and synthetic components. The living organisms regulate the chemical and physical features, resulting in new material properties such as self-regeneration or adaptation to the surrounding environment [235]. Moreover, living organisms can be modified genetically to detect small changes in the environment, and react to them in a programmed manner, e.g. by producing specific enzymes or proteins [236]. For example, Schaffner et al. [237] have proposed a printable hydrogel formulation consisting of HA, κ-carrageenan, and silica loaded with *A. xylinum* bacteria. The chosen bacteria can produce cellulose when exposed to oxygen, in the amount dependent on the oxygen concentration. The results have shown the formation of cellulose after the scaffolds were printed and exposed for oxygen, in a manner dependent on the hydrogel thickness. Higher cellulose production was observed in places where the oxygen concentration was not limited, namely in thicker hydrogel layers. The formation of cellulose after printing led to the mechanically strong constructs. The authors concluded that the scaffolds could play an essential role in skin replacement due to the formation of bacterial cellulose in any required shape [237]. Hybrid living materials were also used to produce agarose scaffolds loaded with *B. subtilis* spores. These spores were engineered to deliver antibiotics to kill the *Staphylococcus aureus*, most often responsible for wound infections. The produced bioink was printed into a patient-specific wound shape. *B. subtilies* in the presence of *Staphylococcus aureus* started secreting an antibacterial lysostaphin or thiocillin. The results indicated that the bioink containing modified bacterium could be used in wound healing, preventing inflammation [238]. We believe that the further development of hybrid living materials will lead to the production of self-sufficient and self-regenerating scaffolds, able to produce the proteins or enzymes necessary for cell proliferation and specific differentiation, which will improve and accelerate tissue regeneration.

### 5.3 4D printing

4D printing uses stimuli-responsive materials to produce dynamic scaffolds [239]. The scaffolds undergo shape transformation or property changes over time in the presence of external stimuli such as magnetic field, light, temperature, or chemical stimuli. Due to the possibility of shape adjustment, dynamic scaffolds can better mimic native tissue behavior and provide a more suitable cell microenvironment [[240], [241], [242], [243]]; the pre-programmed shape scaffolds may fit the defect with higher accuracy compared to the static scaffolds and can be easier to deliver e.g. using laparoscopy [244]. For example, Wang et al. [242] have fabricated NIR-light-sensitive 4D constructs for myocardial regeneration. The 4D scaffold was designed to change shape under light stimulation to better mimic the curved topology of myocardial tissue. This led to uniform cell distribution and enhanced myocardial maturation [242]. The shape-morphing scaffolds were also printed using extrusion bioprinting and MEW for soft tissue engineering. The shape change of methacrylated alginate/PCL scaffolds was triggered by calcium ions, resulting in the programmed scaffold folding. The transformation improved cell proliferation and orientation due to the alignment of cells in the direction of the longer axis of shape-changed scaffolds [243]. We envision that in the future, 4D printing will offer the possibility of developing smart scaffolds with customized sizes and shapes that will change their shape according to tissue regeneration and maturation time. It will also allow the delivery of the scaffolds into hard-to-reach places in the body with minimally invasive surgical strategies.

### 5.4 Metamaterials

Metamaterials are another exciting research area recently benefiting the tissue engineering field. They can be defined as material systems specially engineered to have unusual physical behaviors and distinct mechanical properties that originate from the employed design rather than chemical composition [245]. The Young's modulus, shear modulus, bulk modulus, and Poisson's ratio are carefully optimized to obtain specific compressibility, rigidity, and stiffness of material [246]. Metamaterials are employed to advance tissue regeneration by providing scaffolds with morphology and mechanical properties tailored to the targeted tissue and application [247]. Different designs inspired by the excellent characteristics of metamaterials developed for other engineering fields have been employed to investigate the mechanical performance of multiple human tissues (e.g. bone, skin, muscle, or nerve) [[248], [249], [250], [251]]. The topology features of metamaterials can guide stem cell differentiation and fate [248]. A comprehensive approach for producing metamaterials with a well-defined and controlled architecture is the use of additive manufacturing technologies with the support of simulated models and software programs [252]. We envision combining metamaterials with additional patterned cues for cells will lead to new types of smart and lightweight bio-instructive scaffolds.

### 5.5 Computational modeling

Computer simulations can be a valuable tool to predict how cells react on scaffolds and which scaffold properties are optimal for the specific cells. Koh et al. [253] used modeling to optimize the mechanical properties of scaffolds for cartilage engineering. A finite element method (FEM) model of a 3D knee joint was developed, including bones, cartilage, and the meniscus. Algorithms were used for optimal scaffold design. The results implicated that an optimized scaffold enhances cartilage formation in the knee joint [253]. Another computational modeling approach was used by Tourlomousis et al. [116], who developed a metrology framework based on machine learning. The framework was used to examine the effect of scaffold geometry on the cell shape and focal adhesion proteins distribution on the scaffold. The framework also enabled atomization of image analysis. The obtained data were used as a feed for a machine-learning algorithm classifying various cell phenotypes [116]. The computational models can be of great use for optimizing scaffold properties and predicting material and cell performance before printing. As a result, more time and cost-efficient processing are at hand. Machine learning and artificial intelligence can further increase the potential of computational models by providing continuous improvement and adjustment of the computer-controlled printing process. The computational simulations will play a significant role in accelerating the development of new bio-instructive scaffolds, especially the highly complex ones with multiple cues or hierarchical gradients included. They will assist in optimizing these scaffolds for the desired cell performance.

## 6 Conclusions

EBP of bio-instructive materials is a powerful tool for tissue regeneration. The bio-instructive cues can modulate cell performance by introducing physical or biochemical signals for cells. Employing 3D printing for their deposition allows gaining precise control over those signals’ location and distribution. While one cue may not be sufficient to recapitulate the native tissue microenvironment, the synergy of more cues is a game-changer in gaining full control over directing the cells and clinical translation of bio-instructive materials. We envision that an increasing number of studies will focus on multi-component complex and hierarchical bio-instructive materials. The fabrication of bio-instructive materials containing multiple cues at various levels will push forward the field of tissue engineering and regenerative medicine. Most research in the field to date has been devoted to the development of tissue models, with the focus on bone, cartilage, cardiac, or nerve tissues. The next step is the translation of bio-instructive materials into biomimetic scaffolds, which will be implantable and patient-specific, closely recapitulating the native environment of tissues. It is also expected that the utilization of computer models and machine learning will further increase the potential of the approach and facilitate the clinical translation of bio-instructive scaffolds by providing an optimized match between scaffold properties and desired cellular needs. Biomimicry, 4D printing, and metamaterials approaches, supported by computational modeling and novel manufacturing approaches, will lead to new functional materials, smart designs, and optimized fabrication.

## CRediT authorship contribution statement

**Piotr Stanisław Zieliński:** Conceptualization, Writing – original draft, and, Writing – review & editing, Visualization. **Pavan Kumar Reddy Gudeti:** Writing – original draft, Writing – review & editing. **Timo Rikmanspoel:** Writing – original draft. **Małgorzata Katarzyna Włodarczyk-Biegun:** Conceptualization, Writing – review & editing, Visualization, Supervision, Funding acquisition.

## Declaration of competing interest

None.
